# Supplementary material for: Ultra-high-scale cytometry-based cellular interaction mapping
Source: Nat Methods. 2025 Aug 7;22(9):1887–99. doi: 10.1038/s41592-025-02744-w (PMC12446065; doi:10.1038/s41592-025-02744-w)
Supplement: Supplementary file 1 — Supplementary Figs. 1–5, Notes 1–3 and Refs. [file 41592_2025_2744_MOESM1_ESM.pdf]

nature methods

Supplementary information https://doi.org/10.1038/s41592-025-02744-w

Ultra-high-scale cytometry-based cellular interaction mapping

In the format provided by the authors and unedited

Supplementary Information

Ultra-high scale cytometry-based cellular interaction mapping

Vonficht, Jopp-Saile, Yousefian, Flore et al.

Supplementary Note 1……………………………………………………………………….2

Supplementary Note 2……………………………………………………………………….5

Supplementary Note 3………………………………………………………………………10

Supplement Bibliography……………………………………………………………………14

Supplementary Note 1: Benchmarking of ex vivo cellular interaction mapping

To evaluate the influence of different experimental conditions and the non-specific formation of cellular interactions, we conducted a series of *ex vivo* benchmarking experiments. First, CytoStim^{TM}-treated PBMCs were split, labeled with two distinct fluorescently conjugated CD45 antibodies, reunited, and processed under varying cell concentrations, processing times, and fixation methods (Supplementary Figure 1A-G). Cellular interactions were then mapped using the *Interact-omics* approach. Interactions double-positive for both labels must have been newly acquired during the second incubation, while single-positive interactions could have occurred initially or throughout the culture.

Our findings revealed a robust increase in single-positive interactions following CytoStim^{TM} treatment, whereas double-positive (newly acquired) interactions showed only a mild increase, which was negligible compared to the surge in CytoStim^{TM}-induced single-positive interactions (approximately 5-10 times higher, Supplementary Figure 1G). Extending incubation periods post-CytoStim^{TM} did not significantly elevate newly acquired interactions, though there was a slight decrease in CytoStim^{TM} -induced B-T cell interactions at later time points. These results suggest that *ex vivo*-induced cellular interactions are relatively stable, with newly acquired interactions occurring but having only a minor impact in this context. A mild baseline increase in all interactions was observed with higher cell concentrations, but the relative effect of CytoStim^{TM}-induced single-positive interactions versus newly acquired double-positive interactions remained consistent (Supplementary Figure 1G). Fixation had only a minor effect, with a trend towards higher single- and double- positive interactions upon CytoStim^{TM} treatment in fixed samples (Supplementary Figure 1G).

Collectively, these data provide quantitative insights into how experimental settings impact interactions while demonstrating that *ex vivo* modulations of cellular interactions can be effectively quantified in the explored settings. Besides incubation times, cell numbers and fixation, flow rates and tissue type may significantly impact baseline interactions (Supplementary Figure 1H-I). Therefore, maintaining these parameters constant is critical for maximizing the recovery of signal to background interactions. Notably, cellular interactions were unaffected by cryopreservation

(Supplementary Figure 1J). See Limitations and Guidelines for further details and recommendations.

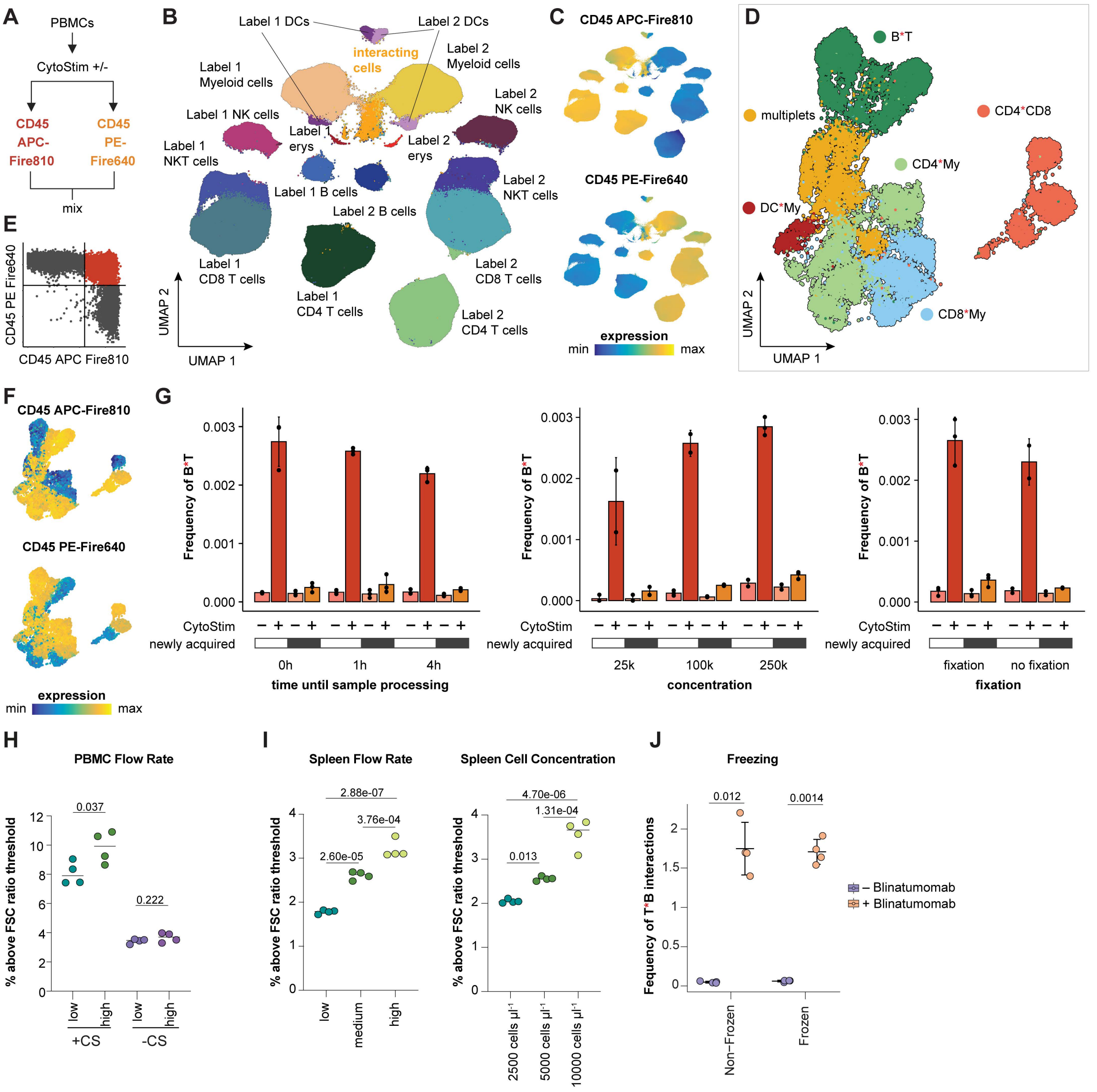

Supplementary Figure 1. Effect of sample processing methods on *ex vivo* cellular interactions. A. Schematic depiction of the experimental approach. Created in BioRender. **B.** Overall cellular landscape across all experimental conditions. n = 3,292,837. Label 1 and 2 refer to CD45 APC-Fire810 and CD45 PE-Fire640, respectively. **C.** Feature plots for the UMAP display in B, colored by the two differently labeled antibodies against CD45. **D.** Interacting landscape across all experimental conditions. n = 23,620. **E.** Dot plot for the CD45 signals in interacting populations, showcasing single-positive and double-positive (highlighted in red) interactions. **F.** Feature plots for the UMAP from panel D, colored by the CD45 signal intensities. **G.** Quantification of single-positive (white) and double-positive, newly acquired (black) interactions in CytoStim™ treated and untreated samples, across different experimental conditions. Left: Varying incubation times at 4°C after mixing, mimicking long sample processing times. Middle: Different cellular concentrations ranging from 25,000 to 250,000 cells in 50 µL during staining and acquisition. Right: Fixation with 2 %

paraformaldehyde after staining compared to no fixation. *n* = 2 to 3 technical replicates. Error bars indicate the standard deviation. **H.** Short-term cultures of PBMCs with or without CytoStim^{TM} measured at low or high flow rate (*n* = 4 technical replicates, the horizontal bar shows the mean). Impact of flow rate on cellular interactions is relatively mild. Two-way ANOVA (CytoStim^{TM}: F(1,13) = 189.138, *P* = 4.01e-09, flow rate: F(1,13) = 6.598, *P* = 0.023), followed by Tukey's Honest Significant Differences test for the flowrate**. I.** Impact of flow rate and cellular concentration on cellular interactions in murine spleens is more pronounced. Left: Baseline interactions in spleens measured with different flow rates (*n* = 4 technical replicates, the horizontal bar shows the mean). Right: Baseline interactions in spleens at different cell densities but constant flow rate (*n* = 4 technical replicates). One-way ANOVA (flow rate: F(2,9) = 115.749, *P* = 3.79e-07, cell concentration: F(2,9) = 61.397, *P* = 5.68e-06), followed by Tukey's Honest Significant Differences tests. **J.** Frequency of T*B cell interactions upon Blinatumomab treatment that were either fixed or fixed after a freeze-thaw cycle (*n* = 4 replicates from a single donor). *P* values were determined with a two-sided Welch's t-test and Bonferroni corrected. Error bars indicate the mean and standard deviation. Abbreviations: UMAP: uniform manifold approximation and projection, CD45 APC-Fire810/CD45-PE-Fire640: fluorescently-coupled CD45 labels, My: myeloid cells, PBMCs: peripheral blood mononuclear cells.

Supplementary Note 2: Benchmarking and interpretation of *in vivo* interaction mapping

To assess the general reliability of interactions derived from *in vivo* settings, we performed ImageStream-based imaging flow cytometry on LCMV-infected spleens on day 7 post-infection. Despite its limitations in cellular throughput and number of measured markers, this method provides morphological information that can be used to distinguish single cells from interacting cells, making it a suitable benchmarking tool. Using a 6-plex panel focused on T and B cell interactions, we first applied the PICtR workflow on the ImageStream data without taking any morphological information into consideration (Supplementary Figure 2A-C). Here, clustering and interaction identification relied solely on fluorescence intensity values as no forward scatter information is recorded with this method. We then compared the results from the *Interact-omics* workflow with data gained from both morphological imaging and/or fluorescence intensity data in order to: (i) differentiate singlets from interacting cells, (ii) identify interacting cell types, and (iii) assess LCMV-induced interaction changes. Comparing the results obtained from the *Interact-omics*-based approach to image segmentation-based classification showed high concordance in singlet and multiplet discrimination (Supplementary Figure 2D, see Methods), further verified by manual inspection of images (Supplementary Figure 2C). Immunophenotypic characterization of interacting populations via conventional gating also showed high agreement with *Interact-omics*-derived annotations (Supplementary Figure 2E). Manual inspection of randomly selected images regarding the localized expression patterns of lineage-specific markers confirmed the expected types of interactions (Supplementary Figure 2C).

Finally, comparing LCMV-induced changes in cellular interactions derived from *Interact-omics* and conventional gating of imaging flow cytometry data, showed a high concordance (Supplementary Figure 2F, G). These findings confirm the *Interact-omics* approach's accuracy in identifying and quantifying single and interacting cell landscapes in case-control settings.

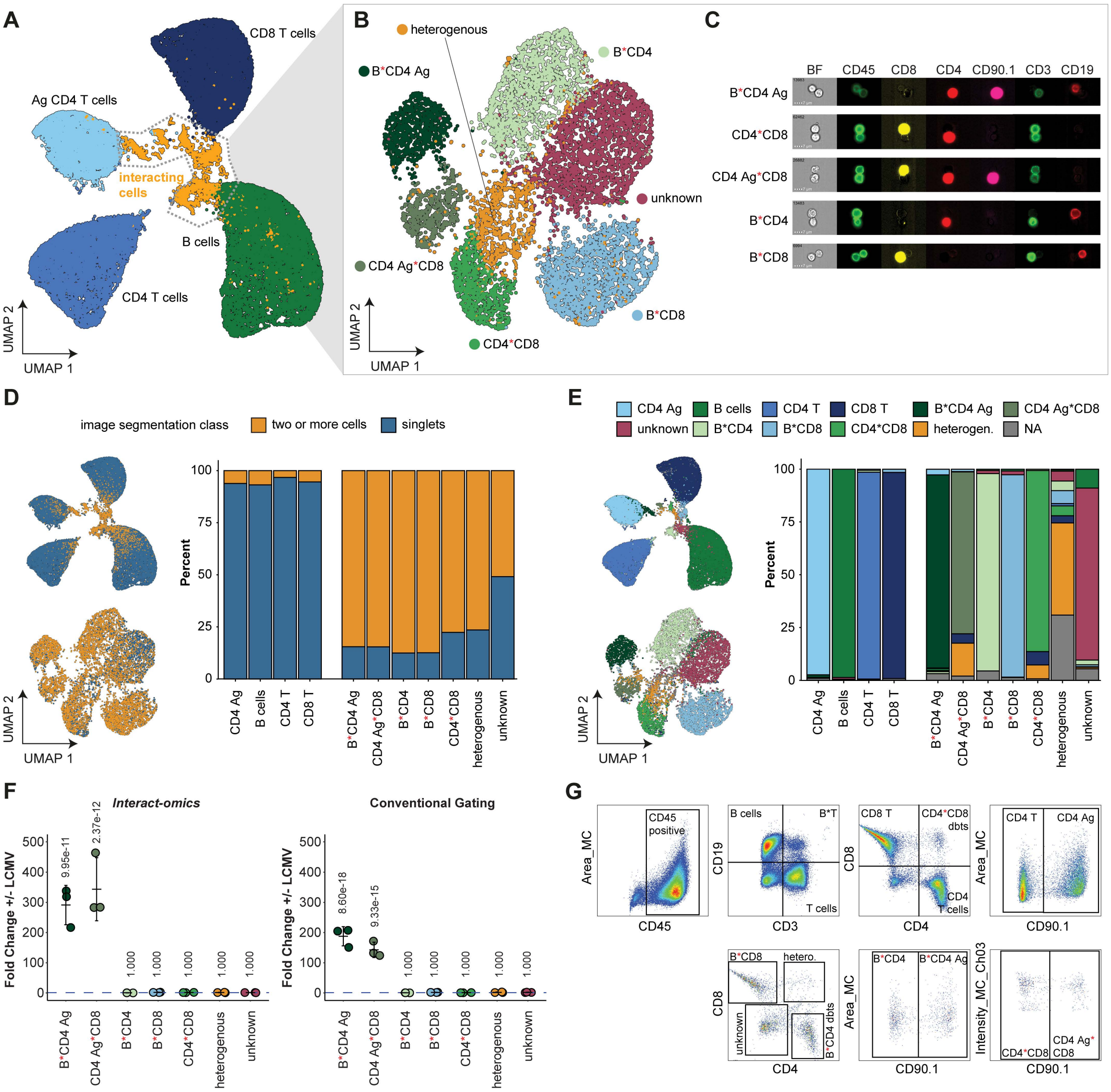

Supplementary Figure 2. Comparison of the *Interact-omics* approach to imaging flow cytometry. A. UMAP representation of the overall cellular landscape derived from the fluorescent intensity values, *n* = 306,538. Intensity values are based on the sum of the pixel intensities in the mask as selected by ImageStream$^{®X}$, background subtracted. The experiment corresponds to day 7 in Supplementary Figure 3. **B.** UMAP representation of the interacting landscape, *n* = 8,683. The heterogeneous cluster is likely mostly comprised of B*CD4*CD8 multiplets. The unknown cluster expresses CD19 and CD3 but no other T cell markers, hindering confident annotation. **C.** Pseudo-colored example images for cellular interactions in the brightfield and fluorescence channels. **D.** Left: UMAP displays from A and B colored by the number of cells identified through image segmentation. Right: Bar plots comparing the populations identified through *Interact-omics* (x-axis labels) and image segmentation (color code). **E.** Left: UMAP displays from A and B colored by populations as identified through conventional gating. Right: Bar plots comparing the populations identified through *Interact-omics* (x-axis labels) and conventional gating (color code). NA indicates that the event does not fall into any conventional gate. **F.** Fold changes of the frequencies +/- LCMV infection. Holm-corrected estimated marginal means comparison. Left: Populations identified by *Interact-omics*. Right: Populations identified through conventional gating. *n* = 3 biological replicates, error bars show the mean and standard deviation. **G.** Gating strategy for

conventional gating. Abbreviations: Ag = antigen-specific, UMAP = uniform manifold approximation and projection, BF = brightfield.

The presented framework measures cellular interactions following sample preparation *ex vivo*. Consequently, for *in vivo* applications, additional cellular interactions may be acquired during sample preparation. While this limitation applies to all cellular interaction mapping approaches that do not rely on specialized mouse models or measure co-localization *in situ*, it remains poorly characterized to what extent this occurs, whether newly acquired interactions are random or directed, and how representative the identified interactions are of the *in vivo* situation.

To evaluate these questions, we utilized congenic mouse models differing in variants of the pan-hematopoietic cell marker CD45, allowing identification of respective immune cells as CD45.1 or CD45.2 using variant-sensitive antibodies (Supplementary Figure 3A). First, we transferred LCMV-specific CD4 T cells (SMARTA: CD90.1-positive, CD45.2-positive) into CD45.2 mice, followed by LCMV infection (group A, Supplementary Figure 3A). Non-infected control CD45.2 mice formed group B. In parallel, we infected CD45.1 mice with LCMV (group C) or left them untreated (group D). On day 7 post-infection, spleens from group A (infected, CD45.2) and group B (non-infected, CD45.2) were either processed individually or mixed with spleens from group C (infected, CD45.1) or group D (non-infected, CD45.1) before tissue homogenization and processing. Applying the *Interact-omics* workflow to these individual and mixed samples resulted in single-cell and interacting cell landscapes of populations that were either single-positive or double-positive for CD45.1 and CD45.2 (Supplementary Figure 3B-E). In this setting, double-positive interactions must have arisen during processing *ex vivo* and can be quantified.

Notably, we observed a substantial fraction of newly acquired double-positive interactions during sample processing (Supplementary Figure 3F). However, newly acquired interactions did not occur randomly but were highly correlated with interactions induced upon infection (Supplementary Figure 3G). In particular, newly acquired interactions in mixed spleens from infected mice compared to non-infected controls were highly correlated with infection-induced single positive interactions in both mixed and non-mixed spleens (Supplementary Figure 3G). This suggests that

while new interactions can be acquired during sample preparation, they are not random but directed and reflect actual biological effects. Notably, a comparison with imaging and *in situ* interaction mapping in an LCMV infection study[1] revealed highly similar interaction types and confirmed key LCMV-induced changes identified by the *Interact-omics* approach, including antigen-specific T cell interactions and transient monocyte-B cell interactions.

Overall, these observations suggest that while it cannot be unequivocally determined whether the measured interactions in the *Interact-omics* approach have all occurred *in vivo*, the interactions are not random but reflect biological effects, and likely are a proxy for cellular interactions occurring *in vivo.* Guidelines for optimizing the approach to avoid potentially misleading results are discussed in the Limitations and Guidelines section.

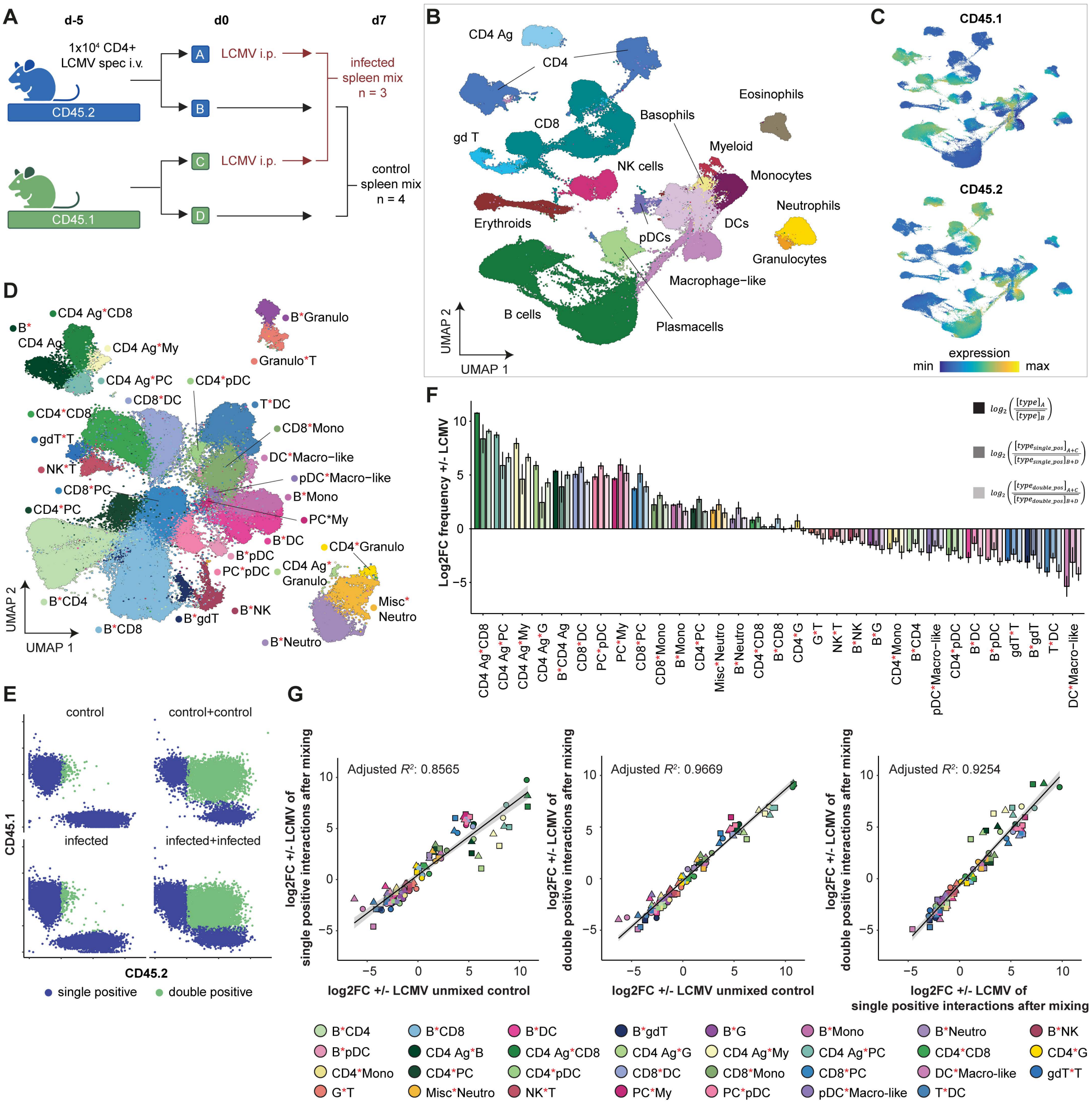

Supplementary Figure 3. Interactions acquired a posteriori after *in vivo* experiment. A. Schematic overview of the experimental approach. LCMV-specific CD4$^+$ T cells were transferred into CD45.2 host mice 5 days before infection with LCMV (group A) or the respective control (group B). Additionally, CD45.1 host mice were infected with LCMV (group C) or left untreated (group D). *n* = 3 for groups A, C, D and *n* = 4 for group B. Created in BioRender. **B.** Single-cell landscape of all experimental groups. Out of *n* = 23,490,812 processed cells, *n* = 245,316 are shown in the UMAP display. **C.** Feature plots for panel B, colored by the expression of the congenital markers CD45.1 and CD45.2. **D.** Interacting landscape across all experimental groups. Out of *n* = 731,621 identifiable interactions, *n* = 93,065 are shown. **E.** Dot plots showcasing the expression of the congenital markers CD45.1 and CD45.2 in interacting populations from the unmixed controls for the untreated and infected conditions, and the mixed spleens from infected mice (infected+infected, group A + group C) or untreated mice (control+control, group B + group D). **F.** Bar plots depicting the log2 fold changes (FC) between the LCMV infected and untreated conditions for each interacting population. Solid bars indicate the log2FC between group A (infected) und group B (control). Semi-transparent bars show the log2FC for single-positive interactions in mixed samples (A+C

for the infected condition, and B+D for the control). Transparent bars depict log2FC between the respective double-positive interactions, which were definitely acquired *ex vivo*. n = 3 mixes across biological replicates, error bars indicate the standard deviation. **G.** Linear relationships between the log2FC between infected and control conditions for unmixed controls, single-positive interactions after mixing and double-positive interactions after mixing. n = 3 mixes across biological replicates. The shaded area shows the 95 % confidence interval for the linear model.

Supplementary Note 3: Application to existing cytometry datasets

To demonstrate the applicability of our approach for analyzing cellular interactions in previously generated datasets, we applied the PICtR workflow to a publicly available cytometry dataset on juvenile idiopathic arthritis (JIA)[2]. JIA is an autoimmune disease characterized by chronic joint inflammation, leading to pain, swelling, and eventual joint damage. While it is hypothesized that abnormal interactions among immune cells – specifically T cells, B cells, and myeloid cells – contribute to the production of inflammatory cytokines and autoantibodies that drive the disease, the precise interaction processes remain poorly understood.

In their study, Attrill and colleagues interrogated PBMC samples from healthy donors, JIA patients with active and inactive disease, and synovial fluid samples from JIA patients with active disease. We downloaded and preprocessed the FCS files as described in the Methods section and ran the flowAI QC algorithm[3] on all FCS files to exclude those with anomalous flow rates from further analysis. High-quality samples were then processed using the PICtR workflow. Notably, we were able to reproduce the single-cell landscape described by Attrill et al., and discovered a range of quantitative and qualitative changes in cellular interactions in the blood of patients with inactive versus active disease, as well as between the blood and synovial fluid of affected joints (Supplementary Figure 4).

Interestingly, in patients with inactive disease, T cells interacting with B cells, predominantly displayed a FoxP3-expressing regulatory T cell phenotype (Supplementary Figure 4I). In contrast, in patients with active disease, these interactions shifted to an inflammatory, non-regulatory phenotype. Similarly, major qualitative differences of interactions between CD4 T cells and monocytes were observed between blood and synovial fluid of patients with active disease (Supplementary Figure 4L). While several of these findings require further validation,

they provide an initial quantitative framework for understanding changes in immune cell interactions that may contribute to disease progression and could help identify targets for therapeutic intervention.

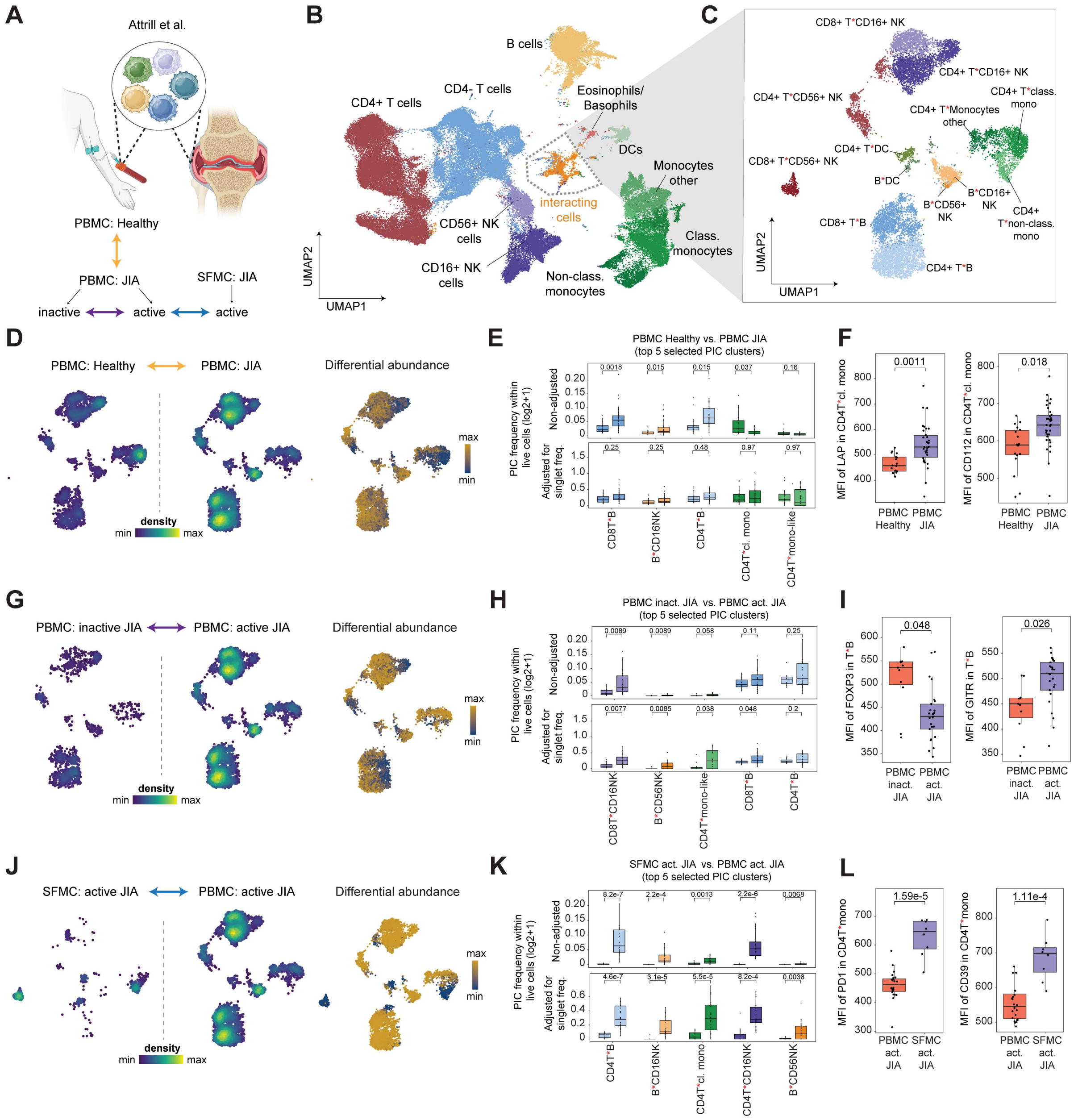

Supplementary Figure 4. Interacting cell landscape in juvenile idiopathic arthritis (JIA). A. Publicly available spectral flow cytometry data of PBMCs and SFMCs of JIA patients[2]. Three comparisons (indicated by the arrows) were made for the interacting cell landscape. Created in BioRender. **B.** UMAP of the overall cellular landscape. Recorded cells were processed with PICtR, out of 7,843,646 cells, 80,000 sketched cells are displayed. **C.** UMAP of interacting cells (*n* = 12,908) **D.** Point density UMAP (left panel) and differential abundance (right panel) of interacting cells comparing PBMCs from healthy donors vs. JIA patients. **E.** Quantitative comparisons of interacting cell frequencies between PBMCs from healthy donors (*n*=18) and JIA patients (*n*=36). Top: Non-adjusted frequencies. Bottom: Interaction frequencies adjusted by the harmonic mean of the singlet frequencies of the contributing cells

(see Methods). *P* values were determined with a two-sided *t*-test and adjusted for multiple testing using Benjamini-Hochberg correction. **F.** Qualitative differences in CD4T*cl.mono interactions. *P* values were determined with a two-sided Wilcoxon rank sum test and adjusted for multiple testing using Benjamini-Hochberg correction. **G.** Point density UMAP (left panel) and differential abundance (right panel) of interacting cells comparing PBMCs of JIA patients with inactive disease (*n*=11) vs. active (*n* =25). **H.** Quantitative comparisons of interacting cell frequencies between PBMCs from JIA with inactive and active disease. Top: Non-adjusted frequencies. Bottom: Interaction frequencies adjusted by the harmonic mean of the singlet frequencies of the contributing cells (see Methods). *P* values were determined with a two-sided t-test and adjusted for multiple testing using Benjamini-Hochberg correction **I.** Qualitative differences in T*B interactions. *P* values were determined with a two-sided Wilcoxon rank sum test and adjusted for multiple testing using Benjamini-Hochberg correction. **J.** Point density UMAP (left panel) and differential abundance (right panel) of interacting cells comparing PBMCs of JIA patients with active disease vs. SFMC of active disease. **K.** Quantitative comparisons of interacting cell frequencies between PBMCs of JIA patients with active disease (*n*=25) vs. SFMC of active disease (*n*=8). Top: Non-adjusted frequencies. Bottom: Interaction frequencies adjusted by the harmonic mean of the singlet frequencies of the contributing cells (see Methods). *P* values were determined with a two-sided t-test and adjusted for multiple testing using Benjamini-Hochberg correction. **L.** Qualitative differences in CD4T*mono interactions. *P* values were determined with a two-sided Wilcoxon rank sum test and adjusted for multiple testing using Benjamini-Hochberg correction. Abbreviations: UMAP = uniform manifold approximation and projection, PBMC = peripheral blood mononuclear cells, SFMC = synovial fluid mononuclear cells. Red asterisks in cell type labels indicate interactions between the respective cell types. Box plots display the median, first and third quartiles and whiskers are defined as 1.5 times interquartile range.

To demonstrate our framework's ability to identify cellular interactions involving non-immune cells, we applied the PICtR workflow to a spectral flow cytometry dataset[4] from the proximal intestine of young and old mice (Supplementary Figure 5A). PICtR identified interactions among $EpCAM^+$ epithelial cells, $Lgr5^+$ intestinal stem cells (ISCs), and various immune cell types (Supplementary Figure 5B, C). In line with previous reports, our analysis confirmed an age-associated increase in CD4 and CD4-CD8 double-positive T cells, a decrease in $CD8^+$ $TCR\alpha\beta^+/TCR\gamma\delta^+$ populations, and a notable increase in interactions between $CD4^+$ $TCR\alpha\beta^+$ T cells and epithelial cells in the aged group (Supplementary Figure 5D, E). The latter supports the reported upregulation of the MHCII machinery in the aged intestine[4], suggesting enhanced antigen-presentation to CD4 T cells.

These analyses demonstrate that our approach can also be applied to existing datasets and non-immune cell types, provided that the data have been generated following the guidelines outlined in this manuscript (see Limitations and Guidelines).

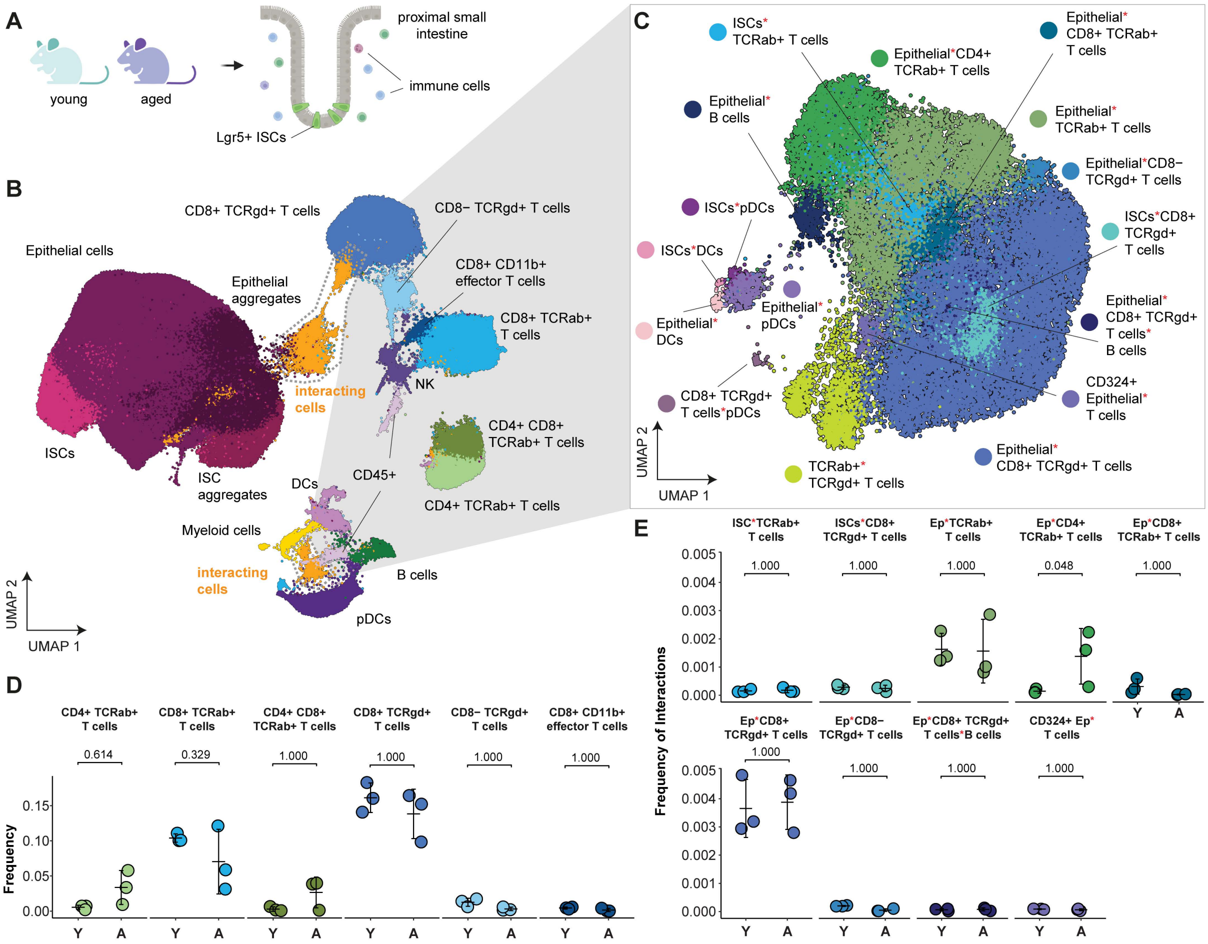

Supplementary Figure 5: Interacting cell landscape in the proximal small intestine. A. Schematic overview of the experimental approach (adapted from Funk and colleagues[4]). *n* = 3 young mice and *n* = 3 aged mice were analyzed. Created in BioRender. **B.** Overall cellular landscape of epithelial cells and the immune microenvironment. Recorded cells were processed with PICtR; out of 4,167,516 cells, 399,608 sketched cells are displayed. **C.** UMAP of the interacting cell landscape, *n* = 32,554. **D.** Comparison of the T cell population frequency in young (*n* = 3) and aged (*n* = 3) mice. *P* values were calculated using least squared means (two-sided) and were Bonferroni-corrected. Error bars indicate the mean and standard deviation. **E.** Comparison of interacting populations that involve T cells in young (*n* = 3) and aged (*n* = 3) mice. *P* values were calculated using least squared means (two-sided) and were Bonferroni-corrected. Error bars indicate the mean and standard deviation.

Supplement Bibliography

1. Sammicheli, S. *et al.* Inflammatory monocytes hinder antiviral B cell responses. *Sci. Immunol.* **1**, (2016).

2. Attrill, M. H. *et al.* The immune landscape of the inflamed joint defined by spectral flow cytometry. *Clin. Exp. Immunol.* (2024) doi:10.1093/cei/uxae071.

3. Monaco, G. *et al.* flowAI: automatic and interactive anomaly discerning tools for flow cytometry data. *Bioinformatics* **32**, 2473–2480 (2016).

4. Funk, M. C. *et al.* Aged intestinal stem cells propagate cell-intrinsic sources of inflammaging in mice. *Dev. Cell* **58**, 2914-2929.e7 (2023).
