# Supplementary material for: Ultra-high-scale cytometry-based cellular interaction mapping
Source: Nat Methods. 2025 Aug 7;22(9):1887–99. doi: 10.1038/s41592-025-02744-w (PMC12446065; doi:10.1038/s41592-025-02744-w)
Supplement: Supplementary file 2 — Reporting Summary [file 41592_2025_2744_MOESM2_ESM.pdf]

nature portfolio

Corresponding author(s): Simon Haas and Daniel Hübschmann

Last updated by author(s): Sep 15, 2024

# Reporting Summary

Nature Portfolio wishes to improve the reproducibility of the work that we publish. This form provides structure for consistency and transparency in reporting. For further information on Nature Portfolio policies, see our Editorial Policies and the Editorial Policy Checklist.

## Statistics

For all statistical analyses, confirm that the following items are present in the figure legend, table legend, main text, or Methods section.

| n/a | Confirmed | |
|---|---|---|
| ☐ | ☒ | The exact sample size (*n*) for each experimental group/condition, given as a discrete number and unit of measurement |
| ☐ | ☒ | A statement on whether measurements were taken from distinct samples or whether the same sample was measured repeatedly |
| ☐ | ☒ | The statistical test(s) used AND whether they are one- or two-sided<br>*Only common tests should be described solely by name; describe more complex techniques in the Methods section.* |
| ☐ | ☒ | A description of all covariates tested |
| ☐ | ☒ | A description of any assumptions or corrections, such as tests of normality and adjustment for multiple comparisons |
| ☐ | ☒ | A full description of the statistical parameters including central tendency (e.g. means) or other basic estimates (e.g. regression coefficient) AND variation (e.g. standard deviation) or associated estimates of uncertainty (e.g. confidence intervals) |
| ☐ | ☒ | For null hypothesis testing, the test statistic (e.g. *F*, *t*, *r*) with confidence intervals, effect sizes, degrees of freedom and *P* value noted<br>*Give P values as exact values whenever suitable.* |
| ☒ | ☐ | For Bayesian analysis, information on the choice of priors and Markov chain Monte Carlo settings |
| ☒ | ☐ | For hierarchical and complex designs, identification of the appropriate level for tests and full reporting of outcomes |
| ☒ | ☐ | Estimates of effect sizes (e.g. Cohen's *d*, Pearson's *r*), indicating how they were calculated |

*Our web collection on statistics for biologists contains articles on many of the points above.*

## Software and code

Policy information about availability of computer code

| | |
|---|---|
| Data collection | For the acquisition of flow cytometry data, SpectroFlo® (Cytek Biosciences, v.3.2.1) or FACSDiva™ (BD, v.8) softwares were used. For imaging cytometry, FacsChorus (v1.3.82) or Cytek® INSPIRE® software (v201.1.0.826) were used. |
| Data analysis | Flow cytometry data was analyzed using FlowJo v10.10.0, PeacoQC for FlowJo v1.5.0 and R 4.3.0. The following packages were used: Seurat v5.0.0, ComplexHeatmap v2.16.0, cytoMEM v1.4.2, MASS v7.3-60, ggplot2 v3.4.4, RColorBrewer v1.1-3, pals v1.8, dplyr v1.1.3, purrr v1.0.2, readr v2.1.4, tibble v3.2.1, tidyr v1.3.0, tidyverse v2.0.0, data.table v1.14.8, rstatix v0.7.2, viridis v0.6.4, khroma v1.11.0, ggpointdensity v0.1.0, clustree v0.5.0,circlize v0.4.15, ggraph v2.1.0, viridisLite v0.4.2, pbapply v1.7-2, ggsci v3.0.0, ggpubr v0.6.0, BPCells v0.1.0, kableExtra v1.3.4, lubridate v1.9.3, forcats v1.0.0, stringr v1.5.0, sp v2.1-1, Spectre v1.0.0, GGally v2.2.0, SeuratObject v5.0.0, R v4.3.0, rpart v4.1.19, mclust 6.1.1, igraph (R) 2.0.3, CellPose 2.2.3, IDEAS 6.2, scikit 0.19.3, Python 3.12.5 , caret 6.0-94, pROC 1.18.5, autothresholdr 1.4.2, bluster 1.15.0,flowCore 2.17.0, CytoML 2.17.0, flowWorkspace 4.17.0, dbscan 1.2-0, flowSOM 2.10.0, flowMeans 1.65.0, Rclusterpp 0.2.6, immunoClust 1.37.111, clue 0.3-65, numpy 2.1.0, pandas 2.2.2, phenograph 1.5.7, leidenalg 0.10.2, matplotlib 3.9.2, seaborn 0.13.2, scipy 1.14.1, ggrepel 0.9.5, igraph (Python) 0.11.6, ggalluvial 0.12.5, PerformanceAnalytics 2.0.4, Hmisc 5.1.-3, corrplot 0.92, cola 2.11.0, e1071 1.7-14, tree 1.0-43, randomForest 4.7-1.1, Bioconductor 3.20, IDEAS® 6.2.189.0, PICtR 0.1.0 / 0.2.1, writexl 1.5.1, FACSDiva v8, SpectroFlo v.3.2.1<br>A detailed description of the data analysis is provided in the Methods.<br>PICtR is available as an open-source R package at github.com/agSHaas/PICtR. Code to reproduce key analysis results can be found at github.com/agSHaas/ultra-high-scale-cytometry-based-cellular-interaction-mapping/. |

For manuscripts utilizing custom algorithms or software that are central to the research but not yet described in published literature, software must be made available to editors and reviewers. We strongly encourage code deposition in a community repository (e.g. GitHub). See the Nature Portfolio guidelines for submitting code & software for further information.

## Data

Policy information about availability of data

All manuscripts must include a data availability statement. This statement should provide the following information, where applicable:

- Accession codes, unique identifiers, or web links for publicly available datasets
- A description of any restrictions on data availability
- For clinical datasets or third party data, please ensure that the statement adheres to our policy

Raw and processed cytometry data for key experiments are provided at doi.org/10.5281/zenodo.10637096. Source data are provided with this paper and can be used to reproduce the analyses and figures in this manuscript.

## Human research participants

Policy information about studies involving human research participants and Sex and Gender in Research.

| | |
|---|---|
| Reporting on sex and gender | Data on sex was collected from patients of the B-ALL cohort. However, given that the patient cohort analyzed here was not part of a clinical trial, sex-specific considerations were not explicitly integrated into the study design. Furthermore, due to the limited sample size of patients with residual disease, a sex-based analysis would undermine the statistical power and reliability of such subgroup analysis and was therefore not performed. Consequently, we do not provide disaggregated sex data but the distribution of male to female participants was balanced (57% male, 43% female). |
| Population characteristics | The median age of patients in the B-ALL study was 9.5 years. Based on the Immuno-phenotype, patients were classified into distinct subgroups: pro B-ALL, pre B-ALL, common B-ALL. or MPAL The majority of patients had a unique karyotype characterized by very specific genetic alterations which included translocations, mutations, deletions, diploidy or haploidy. Due to this extensive heterogeneity, the genetic alterations were not used as covariates in the analysis. |
| Recruitment | Patients received Blinatumomab based on the decision of the treating physicians and recommendations of the national ALL-REZ BFM study center at Charité. This was not part of a clinical trial. Patients were selected for this study according to availability of sample material in the biobank and an informed consent |
| Ethics oversight | Ethics committee of Charité Universitätsmedizin Berlin (reference number: EA2/147/23). |

Note that full information on the approval of the study protocol must also be provided in the manuscript.

# Field-specific reporting

Please select the one below that is the best fit for your research. If you are not sure, read the appropriate sections before making your selection.

☒ Life sciences ☐ Behavioural & social sciences ☐ Ecological, evolutionary & environmental sciences

For a reference copy of the document with all sections, see nature.com/documents/nr-reporting-summary-flat.pdf

# Life sciences study design

All studies must disclose on these points even when the disclosure is negative.

| | |
|---|---|
| Sample size | a) No statistical methods were used to pre-determine the sample size. For mouse experiments, sample sizes were chosen based on the 3R principle, aiming to keep the number of animals to a minimum while obtaining at least 3 biological replicates. For human in-vitro data, experiments were performed with at least three technical replicates.<br>b) Experience suggests that 50 cells are sufficient to robustly identify a cell type and that a given dataset contains roughly 1-5 % of physically interacting cells. The number of cells acquired was sufficient for the analysis. |
| Data exclusions | a) Where applicable, PeacoQC or FlowAI were used to exclude low quality flow cytometry events based on inconsistencies in signal acquisition and speed. Cells were gated accoring to the gating strategy described in the "Flow Cytometry" section. Additionally, cells were removed when high autofluoresence or signal anomalies suggested a low quality event.<br>b) For the B-ALL cohort (n = 42), we compared patients that could unequivocally be categorized into good responders (n = 18) and non-responders (n = 4) in order to explore mechanisms underlying therapy response among patients with residual disease. The remaining 20 patient samples were therefore excluded from the downstream analysis.<br>c) Data points were excluded from the downstream analysis if a population of cells was not detectable across all time points. The excluded populations are noted in the respective figure legends.<br>d) Clusters of physically interacting cells without a cell type exclusive marker combination might represent homotypic interactions and were excluded from the downstream analysis. |
| Replication | All mouse and in-vitro human experiments were performed with at least n = 3 independent or technical replicates and findings could be replicated successfully. |

| | |
|---|---|
| Randomization | Mice, murine samples, and PBMC samples from healthy blood donors were randomly allocated to groups. Randomization is not applicable to the B-ALL cohort, since all patient samples were measured in the presence and absence of Blinatumomab. |
| Blinding | Blinding was not feasible due to the necessity of knowing the treatment/control group allocations for accurate data interpretation and analysis. |

# Reporting for specific materials, systems and methods

We require information from authors about some types of materials, experimental systems and methods used in many studies. Here, indicate whether each material, system or method listed is relevant to your study. If you are not sure if a list item applies to your research, read the appropriate section before selecting a response.

## Materials & experimental systems

| n/a | Involved in the study |
|---|---|
| ☐ | ☒ Antibodies |
| ☒ | ☐ Eukaryotic cell lines |
| ☒ | ☐ Palaeontology and archaeology |
| ☐ | ☒ Animals and other organisms |
| ☒ | ☐ Clinical data |
| ☒ | ☐ Dual use research of concern |

## Methods

| n/a | Involved in the study |
|---|---|
| ☒ | ☐ ChIP-seq |
| ☐ | ☒ Flow cytometry |
| ☒ | ☐ MRI-based neuroimaging |

## Antibodies

Antibodies used

Antibodies (Epitope, Fluorochrome, Vendor, Identifier (RRID), Clone):

Imaging Flow Cytometry:
Anti-CD33 BV421 Biolegend Cat# 303416 WM53
Anti CD19 BV605 Biolegend Cat# 363023 SJ25C1
Anti-HLA-DR BB515 BD Biosciences Cat# 564516 G46-6
Anti-CD3 PE-Cy7 Biolegend Cat# 300419 UCHT1
Anti-CD45 APC Thermo Fisher Scientific Cat# 17-0459-42 HI30
Zombie NIR Biolegend Cat# 423105

CytoStim Experiment:
Anti-CD16 BUV395 BD Biosciences Cat# 563785 3G8
Anti-CD8 BUV496 BD Biosciences Cat# 741199 SK1
Anti-CD33 BUV563 BD Biosciences Cat# 741369 WM33
Anti-CD27 BUV661 BD Biosciences Cat# 741609 M-T271
Anti-CD4 BUV737 BD Biosciences Cat# 612748 SK4
Anti-CD14 BUV805 BD Biosciences Cat# 612902 M5E2
Anti-CD141 BV421 BD Biosciences Cat# 565321 1A4
Anti-CD197 Pacific Blue BioLegend Cat# 353210 G043H7
Anti-CD20 BV480 BD Biosciences Cat# 566132 2H7
Fixable viability dye efluor506 Thermo Fisher Scientific Cat# 65-0866-14
Anti-CD11b BUV605 BD Biosciences Cat# 563015 M1/70
Anti-CD3 CD650 BD Biosciences Cat# 563851 UCHT1
Anti-CD56 BV711 BD Biosciences Cat# 563169 NCAM16.2
Anti-TCRab BV750 BD Biosciences Cat# 747180 IP26
Anti-CD45RA BV785 BD Biosciences Cat# 564552 HI100
Anti-CD45RO FITC Biolegend Cat# 304242 UCHL1
Anti-CD123 PerCP-Cy5.5 BioLegend Cat# 306016 6H6
Anti-CD19 BB700 BD Biosciences Cat# 566396 SJ25C1
Anti-HLA-DR PE Thermo Fisher Scientific Cat# 12-9956-42 LN3
Anti-CD1c PE-Dazzle 594 BioLegend Cat# 331532 L161
Anti-CD154 PE-Cy5 Thermo Fisher Scientific Cat# 16-1541-82 MR1
Anti-CD11c PE-Cy7 BD Biosciences Cat# 561356 B-ly6
Anti-CD45 APC Thermo Fisher Scientific Cat# 17-0459-42 HI30
Anti-CD34 Alexa Fluor700 BD Biosciences Cat# 659123 8G12
Anti-CD69 APC-Cy7 BD Biosciences Cat# 560912 FN50

OT-II Experiment:
Anti-CD19 BUV395 BD Biosciences Cat# 563557 1D3
Anti-CD69 BUV737 BD Biosciences Cat# 612793 H1.2F3
Anti-CD11b BUV805 BD Biosciences Cat# 568345 M1/70
Anti-CD11c BV421 BD Biolegend Cat# 117343 N418
Anti-CD45.1 BV605 BioLegend Cat# 110737 A20
Anti-TCRb BV711 BD Cat# 743002 H57-597

Anti-MHC-II BV786 BioLegend Cat# 107645 2G9
Anti-CD44 FITC eBioscience Cat# 11-0441-82 IM7
Anti-CD86 PE BioLegend Cat# 105105 PO3
Anti-CD4 RY586 BD Biosciences Cat# 568161 GK1.5
Anti-CD14 PE-Cy7 BioLegend Cat# 123316 Sa14-2
Anti-CD25 APC  BioLegend Cat #102053 PC61
Anti-CD8a AF700 eBioscience Cat# 56-0081-82 53-6.7
Anti-CD45.2 APC efluor780 eBioscience Cat# 47-0454 104

CAR-T Experiment:
Anti-CD11c BUV395 BD Biosciences Cat# 564080 HL3
Anti-CD4 BUV496 BD Biosciences Cat# 612952 GK1.5
Anti-CD115 BUV563 BD Biosciences Cat# 748478 CDS-1R
Anti-CD43 BUV615 BD Biosciences Cat# 752307 S7
Anti-CD16/32 BUV737 BD Biosciences Cat# 612783 2.4G2
Anti-MHCII BUV805 BD Biosciences Cat# 748844 M5/114.15.2
Anti-F4/80 SB436 Thermo Fisher Scientific Cat# 562606 BM8
Anti-Ly6G BV480 BD Biosciences Cat# 746448 A18
Fixable viability dye efluor506 Thermo Fisher Scientific Cat# 65-0866-14
Anti-CD3 BV570 Biolegend Cat# 100225 17A2
Anti-CD11b BV605 Biolegend Cat# 101237 M1/70
Anti-CD23 BV650 BD Biosciences Cat# 740456 B3B4
Anti-CD117 BV711 Biolegend Cat# 105835 2B8
Anti-CD150 BV785 Biolegend Cat# 115937 TC15-12F12.2
Anti-CAR GFP
Anti-NK1.1 PerCP Biolegend Cat# 108725 PK136
Anti-CD93 BB700 BD Biosciences Cat# 742187 AA4.1
Anti-SiglecH PerCP efluor710 Thermo Fisher Scientific Cat# 46-0333-82 eBio440c
Anti-CD69 PE Dazzle Biolegend Cat# 104535 H1.2F3
Anti-IgM PE-Cy5 Biolegend Cat# 406544 RMM-1
Anti-CD8a PE-Fire700 Biolegend Cat# 100792 53-6.7
Anti-Ly6C PE-Cy7 Biolegend Cat# 128018 HK1.4
Anti-B220 PE-Fire 810 Biolegend Cat# 103287 RA3-6B2
Anti-CD41 APC Biolegend Cat# 133914 MWReg30
Anti-TCRab AF647 Biolegend Cat# 109218 H57-597
Anti-CD19 SPARK-NIR Biolegend Cat# 115568 6D5
Anti-TCRgd R718 BD Biosciences Cat# 751919 GL3
Anti-CD45 APC-Fire810 Biolegend Cat# 103174 30-F11
Blinatumomab Time Course:
AAnti-CD16 BUV395 BD Biosciences Cat# 563785 3G8
Anti-CD19 BUV496 BD Biosciences Cat# 612938 SJ25C1
Anti-CD33 BUV563 BD Biosciences Cat# 741369 WM53
Anti-CD24 BUV615 BD Biosciences Cat# 751122 ML5
Anti-CD27 BUV661 BD Biosciences Cat# 741609 M-T271
Anti-CD8 BUV737 BD Biosciences Cat# 612754 SK1
Anti-CD45RO BUV805 BD Biosciences Cat# 748367 UCHL1
Anti-CD28 BV421 BD Biosciences Cat# 742525 L293
Anti-CD39 BV480 BD Biosciences Cat# 746454 TU66
Anti-CD71 BV510 BD Biosciences Cat# 743305 M-A712
Anti-CD11c BV605 Biolegend Cat# 301636 3-Sept.
Anti-CD279 BV650 BD Biosciences Cat# 564104 EH12.1
Anti-CD94 BV711 BD Biosciences Cat# 743952 HP-3D9
Anti-TCRab BV750 BD Biosciences Cat# 747180 IP26
Anti-CD45RA BV786 BD Biosciences Cat# 563870 HI100
Anti-Caspase 3/7 probe Thermo Fisher Scientific Cat# C10423
Anti-CD3 Spark Blue Biolegend Cat# 344852 SK7
Anti-CD38 PerCP Biolegend Cat# 303520 HIT2
Anti-CD10 PerCPVio700 Miltenyi Cat# 130-114-5067 REA877
Anti-CD197 PE BD Biosciences Cat# 561008 3D12
Anti-CD56 PE-CF594 BD Biosciences Cat# 564963 R19-760
Anti-CD7 PE-Cy5 Biolegend Cat# 343110 CD7-6B7
Anti-CD25 PE-Fire700 Biolegend Cat# 356145 M-A251
Anti-TCRgd PE-Cy7 BD Biosciences Cat# 655410 11F2
Anti-CD4 PE-Fire810 Biolegend Cat# 344677 SK4
Anti-CD20 APC Biolegend Cat# 302309 2H7
Anti-CD34 APC-R700 BD Biosciences Cat# 659123 8G12
Anti-HLA-DR APC-Cy7 Biolegend Cat# 307618 L243

T Cell Sort:
Anti-CD44 FITC Thermo Fisher Scientific Cat# 11-0441-82 IM7
Anti-CD62L PE-Cy7 BioLegend Cat# 104418 MEL-14
Anti-CD4 APC-Cy7 BioLegend Cat# 100414 GK1.5
Anti-CD8 BUV395 BD Biosciences Cat# 563786 53-6.7

Blinatumomab Treatment:
Anti-CD16 BUV395 BD Biosciences Cat# 563785 3G8

Anti-CD19 BUV496 BD Biosciences Cat# 612938 SJ25C1
Anti-CD33 BUV563 BD Biosciences Cat# 741369 WM53
Anti-CD24 BUV615 BD Biosciences Cat# 751122 ML5
Anti-CD27 BUV661 BD Biosciences Cat# 741609 M-T271
Anti-CD8 BUV737 BD Biosciences Cat# 612754 SK1
Anti-CD45 BUV805 BD Biosciences Cat# 612891 HI30
Anti-CD10 BV421 BD Biosciences Cat# 312218 HI10a
Anti-IgD Pacific Blue Biolegend Cat# 348223 IA6-2
Anti-CD39 BV480 BD Biosciences Cat# 746454 TU66
Anti-CD71 BV510 BD Biosciences Cat# 743305 M-A712
Anti-CD20 PacOrange, BV570 Biolegend Cat# 302331 2H7
Anti-CD11c BV605 Biolegend Cat# 301636 3.9
Anti-CD123 BV650 BD Biosciences Cat# 563405 7G3
Anti-CD56 BV711 Biolegend Cat# 318336 HCD56
Anti-TCRab BV750 BD Biosciences Cat# 747180 IP26
Anti-CD45RA BV786 BD Biosciences Cat# 563870 HI100
Anti-CD57 BB515 BD Biosciences Cat# 565945 NK-1
Anti-CD11b FITC Biolegend Cat# 101206 M1/70
Anti-CD3 SparkBlue Biolegend Cat# 344852 SK7
Anti-CD38 PerCP Biolegend Cat# 303520 HIT2
Anti-CD94 BB700 BD Biosciences Cat# 566534 HP-3D9
Anti-TCRgd PerCP eF710 Invitrogen Cat# 46-9959-42 B1.1
Anti-CD30L PE R&D systems Cat# FAB1028P 116614
Anti-CD279 RY586 BD Biosciences Cat# 568119 EH12.1
Anti-CD1c PE Dazzle Biolegend Cat# 331532 L161
Anti-Tigit PE-Fire 640 Biolegend Cat# 372743 A15153G
Anti-CD25 PE-Fire700 Biolegend Cat# 356145 M-A251
Anti-CD14 PE Cy7 Tonbo Cat# 60-0149-T100 61D3
Anti-CD4 PE-Fire810 Biolegend Cat# 344677 SK4
Anti-CD197 APC BD Biosciences Cat# 566762 2-L1-A
Anti-CD160 AF647 Biolegend Cat# 341203 BY55
Anti-CD69 SPARK-NIR Biolegend Cat# 310957 FN50
Anti-CD127 APC R700 BD Biosciences Cat# 565185 HIL-7R-M21
Anti-CD34 APC-Cy7 Biolegend Cat# 343514 581
Anti-HLA-DR APC-Fire810 Biolegend Cat# 307674 L243

LCMV Experiment:
Anti-MHC-II BUV395 BD Biosciences Cat# 743876 2G9
Anti-Live Dead blue Thermo Fisher Scientific Cat# L34961
Anti-CD24 BUV563 BD Biosciences Cat# 749336 M1/69
Anti-CD11c BUV615 BD Biosciences Cat# 751222 N418
Anti-ICOS BUV737 BD Biosciences Cat# 567919 C398.4A
Anti-CD11b BUV805 BD Biosciences Cat# 568345 M1/70
Anti-CD45.1 BV421 Biolegend Cat# 110732 A20
Anti-CD19 BV480 BD Biosciences Cat# 566167 1D3
Anti-CD117 BV510 Biolegend Cat# 105839 2B8
Anti-Ly6G BV570 Biolegend Cat# 127629 1A8
Anti-CD138 BV605 Biolegend Cat# 142515 281-2
Anti-Ly6C BV650 Biolegend Cat# 128049 HK1.4
Anti-CD90.1 BV711 Biolegend Cat# 202539 OX-7
Anti-CD25 BV785 Biolegend Cat# 102051 PC61
Anti-CD45.2 FITC Biolegend Cat# 109806 104
Anti-CD317 PerCP-efluor710 Biolegend Cat# 127021 927
Anti-CD8 RB780 BD Biosciences Cat# 568692 53-6.7
Anti-CD68 PE Biolegend Cat# 137013 FA-11
Anti-CD4 RY586 BD Biosciences Cat# 568161 GK1.5
Anti-CD172 PE-Dazzle594 Biolegend Cat# 144015 P84
Anti-F480 PE-Cy5 Biolegend Cat# 123111 BM8
Anti-CD357 PE-Cy7 Biolegend Cat# 126309 DTA-1
Anti-TCRb APC Biolegend Cat# 109212 H57-597
Anti-IgD SparkNIR 685 Biolegend Cat# 405749 11-26c.2a
Anti-TCRgd R718 BD Biosciences Cat# 751919 GL3
Anti-CD3 APC-Cy7 BD Biosciences Cat# 561042 145-2C11
Anti-NK1.1 APC-Fire810 Biolegend Cat# 156519 S17016D

Intracellular signaling (revision):
Anti-CD4 Spark UV387 Biolegend Cat# 344686 SK3
Anti-CD3 BUV395 BD Biosciences Cat# 563548 SK7
Anti-CD8 BUV496 BD Biosciences Cat# 741199 SK1
Anti-CD45RO BUV805 BD Biosciences Cat# 748367 UCHL1
Anti-CD19 BV421 Biolegend Cat# 302233 SJ25C1
Anti-CD14 violetFluor 450 Tonbo Cat# 75-0149-T100 61D3
Anti-CD20 BV570 Biolegend Cat# 302331 2H7
Anti-CD56 BV711 Biolegend Cat# 318336 HCD56
Anti-CD45RA BV786 BD Biosciences Cat# 563870 HI100

Anti-CD27 RB705 BD Biosciences Cat# 757295 L128
Anti-Ki67 RB744 BD Biosciences Cat# 570503 B56
Anti-PLCy1 PE Miltenyi Cat# 130-104-969 REA341
Anti-CD94 RY586 BD Biosciences Cat# 753479 HP-3D9
Anti-CD33 PE-Dazzle594 Biolegend Cat# 303431 WM53
Anti-CD197 PE-Fire640 Biolegend Cat# 353261 G043H7
Anti-CD25 PE-Fire700 Biolegend Cat# 356145 M-A251
Anti-TCRgd PE-Cy7 BD Biosciences Cat# 655410 11F2
Anti-pCD247 AF647 BD Biosciences Cat# 558489 K25-407.69
Anti-CD16 cFluor R720 Cytek Biosciences Cat# R7-20006 3G8
Live Dead Zombie NIR Biolegend Cat# 423105
Anti-HLA-DR APC-Cy7 Biolegend Cat# 307618 L243
Anti-CD45 APC-Fire810 Biolegend Cat# 304076 HI30

Imaging flow cytometry (revision):
Anti-CD45.2 RB545 BD Biosciences Cat# 756290 104
Anti-CD8 PE BioLegend Cat# 100707 53-6.7
Anti-CD4 Pe-Fire640 BioLegend Cat# 100481 GK1.5
Anti-CD90.1 Pe-Cy7 BioLegend Cat# 202518 OX-7
Anti CD45.1 BV421 BioLegend Cat# 110732 A20
Anti-CD3 BV510 BioLegend Cat# 100234 17A2
Anti-CD19 SPARK-NIR 587 BioLegend Cat# 115568 6D5

LCMV experiment (revision):
Anti-CD19 Spark UV 387 Biolegend Cat# 115585 6D5
Anti-CD48 BUV395 BD Biosciences Cat# 740236 HM48-1
Anti-CD4 BUV496 BD Biosciences Cat# 612952 GK1.5
Anti-CD44 BUV563 BD Biosciences Cat# 741227 IM7
Anti-CD43 BUV615 BD Biosciences Cat# 752307 S7
Anti-CD71 BUV661 BD Biosciences Cat# 741481 C2
Anti-CD24 BV737 BD Biosciences Cat# 612832 M1/69
Anti-CD62L BUV805 BD Biosciences Cat# 741924 MEL-14
Anti-CD45.1 BV421 Biolegend Cat# 110732 A20
Anti-SiglecF SB436 Thermo Fisher Scientific Cat# 62-1702-82 1RNM44N
Anti-CD105 Pacific Blue Biolegend Cat# 120411 MJ7/18
Anti-Ly6G BV480 BD Biosciences Cat# 746448 A18
Anti-CD3 BV510 Biolegend Cat# 100234 17A2
Anti-NK1.1 BV570 Biolegend Cat# 108733 PK136
Anti-CD172a BV605 BD Biosciences Cat# 740390 P84
Anti-CD23 BV650 BD Biosciences Cat# 740456 B3B4
Anti-CD117 BV711 Biolegend Cat# 105835 2B8
Anti-CD138 BV785 Biolegend Cat# 142534 281-2
Anti-CD45.2 RB545 BD Biosciences Cat# 756290 104
Anti-CD21/35 APC BD Biosciences Cat# 123412 7E9
Anti-Ly6C PerCP Biolegend Cat# 128028 HK1.4
Anti-CD317 BB700 BD Biosciences Cat# 747601 927
Anti-IgM PerCP-efluor710 Thermo Fisher Scientific Cat# 46-5790-80 II/41
Anti-CD11b RB744 BD Biosciences Cat# 570513 M1/70
Anti-MHCII PE Tonbo Cat# 50-5321-U100 M5/114.15.2
Anti-F4/80 Spark YG 593 Biolegend Cat# 157311 QA17A29
Anti-CD64 PE-Dazzle594 Biolegend Cat# 164412 W18349C
Anti-CD25 PE-Fire640 Biolegend Cat# 102071 PC61
Anti-CD11c PE-Cy5 Biolegend Cat# 117316 N418
Anti-CD8a PE-Fire700 Biolegend Cat# 100792 53-6.7
Anti-CD90.1 PE-Cy7 Biolegend Cat# 202518 OX-7
Anti-B220 PE-Fire810 Biolegend Cat# 103287 RA3-6B2
Anti-CD41 APC Biolegend Cat# 133914 MWReg30
Anti-TCRab AF647 Biolegend Cat# 109218 H57-597
Anti-TCRgd R718 BD Biosciences Cat# 751919 GL3
Live Dead Zombie NIR Biolegend Cat# 423105
Anti-Sca1 APC-Cy7 BD Biosciences Cat# 560654 D7

Validation

All antibodies used in this study are commercially available, broadly established, and validated by the respective manufacturers for the indicated species and applications, as detailed on their websites (see RRIDs above for each antibody). Validation information for each primary antibody includes species reactivity, specificity, and application data provided by the manufacturers.

In addition, all primary antibodies have been routinely used in our laboratory with reproducible and consistent results across multiple experiments and independent batches. This includes verification of expected staining patterns in positive control tissues/cells and the absence of non-specific staining in negative controls

# Animals and other research organisms

Policy information about studies involving animals; ARRIVE guidelines recommended for reporting animal research, and Sex and Gender in Research

| | |
|---|---|
| Laboratory animals | Mice were maintained in individually ventilated cages under SPF conditions in the animal facility of the DKFZ (Heidelberg, Germany) or at the Charité animal facility (FEM, Berlin, Germany) with ad libitum access to water and food (22 ± 2 °C, 45-65 % humidity, 12h light-dark cycle). Mice used in LCMV experiments were 7 weeks old; all other mice were between 6-20 weeks old. CD45.1 mice were obtained from in house breeding at DKFZ (Z110I02, B6.SJL- Ptprca Pepcb/BoyJ) or from Charles Rivers (B6.SJL-PtprcaPepcb/BoyCrl). For experiments with antigen-specific T cells, cells were isolated from B6.Cg-Tg(TcraTcrb)425Cbn/J (OT-II) or LCMV-TCRtg P1454 and Smarta55 mice expressing the congenic markers CD45.1 or CD90.1. All other mice were C57BL6/J. |
| Wild animals | The study did not involve wild animals. |
| Reporting on sex | All mice were female. |
| Field-collected samples | No samples were collected from the field. |
| Ethics oversight | Unless otherwise stated, animal experiments were conducted under German law and approved by Regierungspräsidium Karlsruhe (approval number DKFZ299, G-55/20, G-56/20) or the Landesamt für Gesundheit und Soziales in Berlin (LAGeSo, G0016/20). |

Note that full information on the approval of the study protocol must also be provided in the manuscript.

# Flow Cytometry

## Plots

Confirm that:

☒ The axis labels state the marker and fluorochrome used (e.g. CD4-FITC).

☒ The axis scales are clearly visible. Include numbers along axes only for bottom left plot of group (a 'group' is an analysis of identical markers).

☒ All plots are contour plots with outliers or pseudocolor plots.

☒ A numerical value for number of cells or percentage (with statistics) is provided.

## Methodology

Sample preparation

a) PBMC samples from healthy blood donors were obtained as buffy coats from the blood donation center IKTZ Heidelberg. Mononuclear cells were isolated by Ficoll (GE Healthcare) density gradient centrifugation and stored in FCS 10% DMSO in liquid nitrogen until usage. Cryopreserved PBMCs were thawed in a water bath at 37°C, transferred to 10% FCS RPMI-1640 and washed twice. After each washing step, cells were centrifuged at 350g for 5 min. 2 × 105 cells were plated in 10% FCS RPMI-1640 and cultured short term for up to 5h in 200 µl RPMI 10% FCS. CytoStim (Miltenyi) was used in concentrations recommended by the manufacturer at 37°C for 2 hours before harvest. For experiments using a Blinatumomab analog (Invivogen), a concentration of 50 ng/ml was used. The incubation period ranged from 0.25 to 5 hours at 37°C and 5% CO2 in 96-well U-bottom plates. For experiments assessing the stability of Blinatumomab-induced interactions upon cryopreservation, cells were either incubated for 2h in presence of the compound and stained with surface antibodies and fixed with 4% PFA (ThermoFisher) or frozen in Bambanker freezing medium (Nippon Genetics), thawed after 18h and treated in the same way as the non-frozen cells.

b) Blinatumomab response analysis:  Bone marrow aspirates obtained from 42 relapsed B-ALL patients were thawed in a water bath at 37°C, transferred to 10% FCS RPMI-1640 and washed twice. After thawing, each sample was split into two. One half of the sample was cultured in 200 µl RPMI 1640 (10% FCS) supplemented with 50ng/ml Blinatumomab analog (Invivogen) for 1 hour at 37°C and 5% CO2 in a 96 well U-bottom plate. The other half of the sample was cultured in RPMI 1640 (10% FCS) without Blinatumomab supplementation for 1h at the same conditions. After the incubation, cells were harvested, washed with FACS buffer, stained with the surface marker panel and analyzed.

c) For in vitro benchmarking experiments, human PBMCs were treated with CytoStimTM as described above; control groups were left untreated. Subsequently, cells were split into two groups each and stained with CD45-APC-Fire810 or CD45-PE-Fire640, respectively. After mixing the labelled groups, cells were incubated for 0-4h at 4 °C (200 000 cells/well in 50 µL during staining/acquisition) or processed at seeding densities of 25,000 to 250,000 cells per well in 96-well plates (50 µL during staining/acquisition). Subsequently, cells were harvested, washed with FACS-buffer, stained with surface markers, fixed with 2 % PFA (except the non-fixed control) and analyzed.

d) For measuring phosphorylated CD247, human PBMCs were seeded at 100,000 cells/well in 200 µL and treated for 1h with Blinatumomab (160 ng/mL) or CytoStimTM as described above. Following the stimulation period, cells were fixed immediately by adding CytoFix buffer (15 min, 4°C). Cells were washed and resuspended in 200 µL 2.5x Perm/Wash buffer, incubated for 30 min at 37°C, and stained overnight at 4°C before analysis.

e) Mice were euthanized through cervical dislocation. For isolation of antigen-specific T cells, the spleen and various lymph nodes (including inguinal, axial, submandibular, and mesenteric) were carefully extracted. Tissues were homogenized using a 40µm filter (Falcon) and a syringe plunger in cold RPMI (Sigma Aldrich) with 2% FCS (Gibco by Lifetechnologies).

Subsequently, single-cell suspensions from spleens were treated with erythrocyte lysis solution (ACK buffer, containing 0.15 M $NH_4Cl$, 1 mM $KHCO_3$, and 0.1 mM $Na_2EDTA$ in water from Lonza) for a duration of 5 minutes. For some readouts, these suspensions were combined with the lymph node samples or maintained separated. CD4+ and CD8+ T cells were purified using either the Dynabeads Untouched Mouse CD4 Cells Kit (Invitrogen) or the murine CD4+ T cell isolation kit and the murine CD8+ T cell isolation kit (Miltenyi) according to the manufacturer's instructions. Purified fractions were stained for further purification using FACS (see section Flow cytometry, cell sorting and image cytometry). For in vivo experiments, femurs, spleen and various lymph nodes were dissected and kept separate on ice. Lymph nodes and spleens were individually processed as described above. Femurs were flushed using FACS buffer and homogenized using a 40µm filter (Falcon) and a syringe plunger.

f) For the in vivo benchmarking experiment, LCMV-specific CD4+ T cells were transferred into C57BL6 (CD45.2) hosts 5 days prior to infection as described above. CD45.1 (B6.SJL-PtprcaPepcb/BoyCrl) and CD45.2 hosts were infected intraperitoneally as described above, and spleens were harvested on day 7 post-infection. Spleens were split into 4 equal pieces and mixed across CD45.1/CD45.2 hosts for joint tissue homogenization (see Supplementary Figure 11A). Mixed samples were processed for spectral flow cytometry analysis.

Unless otherwise stated, cell suspensions were resuspended in 2% FSC 0,5 mM EDTA PBS (FACS buffer) for performing flow cytometric stainings. For ex vivo readouts with bi-specific engagers and antigen specific T cells, cells were harvested, centrifuged 5 min at 350 g and stained with surface marker panel master mixes using FACS buffer and addition of Brilliant Stain buffer (BD) according to the manufacturer's recommendation. Cells were stained for 30 min on ice in 96-well V-bottom plates, followed by washing with FACS buffer, centrifugation for 5 min at 350g and resuspension in 200 µl FACS buffer. For more time-consuming in vivo experiments, cells were labeled with fixable dead cell exclusion dyes followed by fixation of obtained single-cell suspensions with cold 2% PFA PBS for 15 min at room temperature. Cells were washed, centrifuged for 5 min at 350 g and then stained for 12h at 4°C. After washing and centrifugation for 5 min at 350g, cells were filtered through a 35-µm cell strainer and kept on ice until flow cytometric analysis.

For image-enabled cell sorting, PBMCs were incubated for 2h with CytoStim, stained with surface markers followed by fixation with 2% PFA PBS as described above and operated using a 100 µm sort nozzle, with the piezoelectric transducer driven at 34 kHz and automated stream setup by BD FACSChorusTM Software, and a system pressure of 20 psi.

For the ImageStream®X experiment, data was acquired using the Cytek® INSPIRE™ software. ImageStream®X fluorescence intensity values (based on the sum of the pixel intensities in the mask as selected by ImageStream®X, background subtracted) were compensated and transformed using FlowJo (v10.10) and IDEAS (v6.2). Data was processed using PICtR (see below). Interacting populations were solely annotated based on mutually exclusive marker expression, since forward scatter properties are not acquired by ImageStream®X. For conventional gating, gates were selected in FlowJo according to the strategy shown in Supplementary Figure 10G.

Instrument

For flow cytometric analysis, a Cytek Aurora (Cytek Biosciences) or LSR Fortessa (BD) equipped with 5 lasers were used. For sorting of naive T cells in ex vivo setups, FACSAria Fusion or FACSAria II sorters equipped with 70 µm nozzles were used. For imaging cytometry, image-enabled cell sorting using the BD CellViewTM Imaging Technology was used. For the ImageStream®X experiment, data was acquired using the ImageStream®X (Cytek) and the Cytek® INSPIRE™ software.

Software

Software used for the acquisition and analysis of flow cytometry and image cytometry data is described above in the "Software and Code" section.

Cell population abundance

Purity in post sort fractions was not directly determined. Post sort cytometry data gave detailed insights into the biology of sorted cell populations.

Gating strategy

FSC-SSC gates were set so that FSC-low and SSC-high events were excluded (cell gate). If applicable, dead cells were removed by gating on cells low in viability dyes (Anti-Live Dead blue, efluor506, or Zombie NIR).

☒ Tick this box to confirm that a figure exemplifying the gating strategy is provided in the Supplementary Information.
