# Supplementary material for: Ultra-high-scale cytometry-based cellular interaction mapping
Source: Nat Methods. 2025 Aug 7;22(9):1887–99. doi: 10.1038/s41592-025-02744-w (PMC12446065; doi:10.1038/s41592-025-02744-w)
Supplement: Supplementary file 5 — PICtR source code repository. [file 41592_2025_2744_MOESM5_ESM.zip › PICtR-main/PICtR_1.0.0.pdf]

Package ‘PICtR’

January 16, 2025

Type Package

Title Physically Interacting Cell (PIC) toolkit for R

Version 1.0.0

Date 2025-01-16

Description This is the computational workflow to analyse physically interacting cells (PICs) in flow cytometry data using R.

URL https://github.com/agSHaas/PICtR

BugReports https://github.com/agSHaas/PICtR/issues

License file LICENSE

Depends R (>= 4.3),
Seurat (>= 5.0.1),
dplyr (>= 1.1.4),
ggplot2 (>= 3.5.0)

Imports BPCells (>= 0.1.0),
MASS,
SeuratObject (>= 5.0.1),
cowplot,
data.table,
ggrepel,
khroma (>= 1.12.0),
pals (>= 1.8),
pbapply,
readr (>= 2.1.5),
scales,
tibble,
tidyr (>= 1.3.1)

Suggests Spectre (>= 1.1.0),
ComplexHeatmap (>= 2.16.0),
cytoMEM (>= 1.4.0),
ggrastr,
autothresholdr (>= 1.4.2),
dbscan,
flowMeans,
knitr,
rmarkdown,
testthat

VignetteBuilder knitr

Encoding UTF-8

LazyData true

RoxygenNote 7.3.1

Config/testthat/edition 3

Contents

calculateThreshold 2
demo_lcmv 3
MEM_heatmap 4
predict_data 5
ratio_cluster_plot 6
save.obj 6
save.plot 7
select_dbt 7
sketch_wrapper 8
split_plot_sketch 10
umap_rasterized 11
wrapper_for_plots 11

Index **13**

`calculateThreshold` *Threshold calculation*

Description

Calculates a threshold based on different methods.

Usage

```
calculateThreshold(data, method = "otsu", breaks = 2000, seed = 42)
```

Arguments

`data`	The data to calculate a threshold for. Must be non-negative integers for autothresholdr methods, see below.
`method`	The method used for thresholding. One of: "otsu", "triangle", "kmeans" or methods from the autothresholdr package, see `auto_thresh` ("IJDefault", "Huang", "Huang2", "Intermodes", "IsoData", "Li", "MaxEntropy", "Mean", "MinErrorI", "Minimum", "Moments", "Otsu", "Percentile", "RenyiEntropy", "Shanbhag", "Triangle" and "Yen"). For autothresholdr methods, the data will be forced to integers (Default: "otsu").
`breaks`	Number of histogram breaks for methods that rely on histograms. Should be >100 (Default: 2000).
`seed`	Seed for reproducibility (Default: 42).

Value

Numerical threshold value

References

N. Otsu, "A Threshold Selection Method from Gray-Level Histograms," in IEEE Transactions on Systems, Man, and Cybernetics, vol. 9, no. 1, pp. 62-66, Jan. 1979, doi: 10.1109/TSMC.1979.4310076.

Zack GW, Rogers WE, Latt SA. Automatic measurement of sister chromatid exchange frequency. Journal of Histochemistry & Cytochemistry. 1977;25(7):741-753. doi: 10.1177/25.7.70454.

Hartigan, J. A., & Wong, M. A. (1979). Algorithm AS 136: A K-Means Clustering Algorithm. Journal of the Royal Statistical Society. Series C (Applied Statistics), 28(1), 100–108. https://doi.org/10.2307/2346830.

G. Landini, D.A. Randell, S. Fouad, and A. Galton. Automatic thresholding from the gradients of region boundaries. Journal of Microscopy, 265(2), 185-195.

Examples

```
data("demo_lcmv")
demo_lcmv$ratio <- demo_lcmv$FSC.A/demo_lcmv$FSC.H
threshold <- calculateThreshold(demo_lcmv$ratio, method = "otsu", breaks = 2000)
```

`demo_lcmv` *Demo data set*

Description

Demo data set for the PICtR workflow containing sampled cells from the spleens of LCMV infected mice (day 7) and control. For demonstration purposes, only CD3, MHCII, CD11c, CD11b, CD45_2, CD19, Ly6G, CD90_1, CD4, and CD8 are included as cell type markers.

Usage

```
data(demo_lcmv)
```

Format

72620 observations (cells) and 14 variables (markers, scaled to 0-1023).

FSC.A Forward scatter area

FSC.H Forward scatter height

SSC.A Side scatter area

SSC.H Side scatter height

fluorescence markers CD3, MHCII, CD11c, CD11b, CD45_2, CD19, Ly6G, CD90_1, CD4, CD8

References

Vonficht, Jopp-Saile, Yousefian, Flore et al. Ultra-high scale cytometry-based cellular interaction mapping. Nature Methods (2025)

MEM_heatmap *Marker Enrichment Modeling (MEM) Heatmap.*

Description

Marker Enrichment Modeling (MEM) Heatmap.

Usage

```
MEM_heatmap(
  obj,
  markers = c(),
  cluster_col = "seurat_clusters",
  cols = pals::coolwarm(100),
  heatmap_name = "MEM enrichment score",
  heatmap_column_title = "marker",
  heatmap_row_title = "cluster",
  scale_width = 2.2,
  scale_height = 5
)
```

Arguments

`obj`	The Seurat object.
`markers`	Meta.data columns with features that should be plotted in the heatmap and the clustering resolution.
`cluster_col`	Character string specifying the column that contains the clustering solution.
`cols`	Color palette.
`heatmap_name`	Title of the heatmap.
`heatmap_column_title`	Title for the columns of the heatmap.
`heatmap_row_title`	Title for the rows of the heatmap.
`scale_width`	Scaling factor for the width of the heatmap in relation to the number of columns.
`scale_height`	Scaling factor for the height of the heatmap in relation to the number of rows.

Value

MEM heat map.

predict_data *Cluster label prediction*

Description

Predicts the cluster labels for a reference data set to a query data set using lda.

Usage

```
predict_data(
  obj = obj,
  data_query = query,
  ref_clusters = NULL,
  FSC.A = "FSC.A",
  FSC.H = "FSC.H",
  pred_name = "clusters_predicted",
  assay_ref = NULL,
  assay_query = NULL,
  chunk_size = 1e+06,
  return_obj = TRUE
)
```

Arguments

obj	The Seurat object.
data_query	A data frame or a Seurat object with the cells whose labels should be predicted.
ref_clusters	The meta.data column with the reference cluster labels.
FSC.A	The name of the column containing the FSC.A scatter parameter.
FSC.H	The name of the column containing the FSC.H scatter parameter.
pred_name	The name of the meta.data column for predicted cluster labels (character vector).
assay_ref	The name of the Seurat assay which was used to calculate the reference cluster labels.
assay_query	The name of the Seurat assay containing cells whose labels should be predicted. Only if the query is provided as a Seurat object.
chunk_size	Chunk size for the prediction progress for verbose output to standard out.
return_obj	Boolean. Add the predicted cluster labels to the Seurat object? Only if the query is provided as a data frame.

Value

Seurat object or data frame containing the predicted cluster labels.

References

Venables, W. N. & Ripley, B. D. (2002) Modern Applied Statistics with S. Fourth Edition. Springer, New York. ISBN 0-387-95457-0

Ripley, B. D. (1996) Pattern Recognition and Neural Networks. Cambridge University Press.

ratio_cluster_plot *Ratio cluster plot.*

Description

Stacked bar plot of each cluster with the proportion of cells below/above the threshold determined with Otsu's method using the FSC.A/FSC.H ratio.

Usage

```
ratio_cluster_plot(
  obj,
  clusters = "seurat_clusters",
  ratio = "ratio_anno",
  assay = "FACS"
)
```

Arguments

obj	The Seurat object.
clusters	The string of the meta.data column with the clustering resolution to plot.
ratio	The meta.data column with the classification of cells (ratio_high/ratio_low) determined using the FSC.A/FSC.H ratio and the determined threshold using Otsu's method.
assay	The Seurat assay to use (default FACS).

Value

None

save.obj *Save Seurat object*

Description

Wrapper function for saving Seurat objects

Usage

```
save.obj(object, path = output_obj_path, file)
```

Arguments

object	The Seurat Object.
path	Directory path.
file	File name.

Value

None

save.plot — *Save plot*

Description

Wrapper function for saving plots as a pdf file.

Usage

```
save.plot(plot, path = output_plot_path, file, dim_h = 7, dim_w = 5)
```

Arguments

plot	The plot.
path	Directory path.
file	The file name.
dim_h	Height parameter for pdf
dim_w	Width parameter for pdf

Value

None

select_dbt — *Selection of clusters containing physically interacting cells.*

Description

Selects the clusters that belong to a given percentile of the clusters with the largest physically interacting cells proportion based on FSC ratio thresholding. By default, the top 20 percent of clusters are chosen. Consider using ratio_cluster_plot to evaluate the choice of percentile cutoff.

Usage

```
select_dbt(
  obj,
  clusters = "seurat_clusters",
  ratio = "ratio_anno",
  ratio_high = "Ratio_high",
  assay = "FACS",
  quantile = 0.8,
  selected_clusters = "doublet_clusters"
)
```

Arguments

- `obj` The Seurat object.
- `clusters` The meta.data column containing the cluster labels (Default: seurat_clusters).
- `ratio` The meta.data column with the classification of cells into ratio_high and ratio_low using the FSC ratio (FSC.A/FSC.H) and a thresholding method.
- `ratio_high` The character string that indicates cells with a FSC ratio (FSC.A/FSC.H) above the threshold determined with `calculateThreshold` within the meta.data column.
- `assay` The Seurat assay containing FACS data.
- `quantile` The desired percentile cutoff above which clusters are classified as physically interacting cell clusters (Default 0.8, meaning top 20 percent of clusters are chosen).
- `selected_clusters` Character vector for the misc slot in the Seurat object that will contain the cluster numbers of the selected physically interacting cell clusters (Default: "doublet_clusters").

Value

Seurat object

`sketch_wrapper` *PICtR workflow wrapper*

Description

Characterizes cells into ratio_high and ratio_low cells for physically interacting cell analysis, samples a representative subset of cells using `SketchData` and runs the standard Seurat analysis workflow.

Usage

```
sketch_wrapper(
  channel = channel,
  meta_data = NULL,
  assay = "FACS",
  FSC.A = "FSC.A",
  FSC.H = "FSC.H",
  n_sketch_cells = 50000,
  n_components = "all",
  clst_algorithm = 1,
  resolution = c(0.5, 1, 2, 3, 4),
  min_clst_size = 100,
  meta.k = "auto",
  obj_name = "obj_sketched_non_projected",
  ratio = TRUE,
  thresholding_method = "otsu",
  hist_breaks = 2000,
  group_by = NULL,
```

```
  verbose = TRUE,
  BPcell_dir = NULL,
  overwrite = F,
  working_dir = getwd(),
  seed = 42
)
```

Arguments

channel	A data frame with the dimensionality cells x flow cytometry parameters.
meta_data	A data frame with meta_data for every cell (Default=NULL).
assay	A character string with the name of the assay to be created (Default="FACS").
FSC.A	The name (string) of the column containing the FSC.A scatter parameter (Default="FACS.A").
FSC.H	The name (string) of the column containing the FSC.H scatter parameter (Default="FACS.H").
n_sketch_cells	The number of cells to be subsampled by SketchData (Default=50000).
n_components	The number of components computed and used during analysis. "all" or given as an integer (Default="all").
clst_algorithm	Algorithm used for clustering. For Seurat's FindClusters: 1 = original Louvain algorithm; 2 = Louvain algorithm with multilevel refinement; 3 = SLM algorithm; 4 = Leiden algorithm). Leiden requires the leidenalg python. Alternatively, "hdbscan" for Hierarchical Density-Based Spatial Clustering of Applications with Noise (HDBSCAN) on the UMAP embedding, see hdbscan), "flowMeans" for non-parametric clustering and segmented-regression-based change point detection, see flowMeans, or "flowSOM" for self-organizing maps, see run.flowsom, (Default: Louvain, 1)
resolution	A numerical vector with the desired resolutions for clustering (Default: c(0.5,1,2,3,4)). Only used for Louvain, SLM, and Leiden clustering.
min_clst_size	minimum cluster size for HDBSCAN. See details at hdbscan (Default: 100).
meta.k	meta k for FlowSOM clustering, see run.flowsom (Default: "auto").
obj_name	The name used for storing the Seurat object (character).
ratio	Boolean variable. If TRUE the FSC ratio (FSC.A/FSC.H) will be calculated.
thresholding_method	Method for thresholding. One of "otsu", "triangle", "kmeans", or any method from the autothresholdr package. For details see ?calculateThreshold() (Default = "otsu").
hist_breaks	Number of histogram breaks for methods that rely on histograms. Should be >100 (Default: 2000).
group_by	Optional grouping parameter to calculate the FSC ratio (FSC.A/FSC.H) thresholding method for.
verbose	Verbosity (Boolean).
BPcell_dir	Optional directory with the counts matrix for open_matrix_dir.
overwrite	Overwrite existing BPCells directory? (Default: FALSE)
working_dir	Directory path to be used as working directory (character string).
seed	Seed for reproducibility (Default: 42).

Value

Seurat object.

References

Hao et al. Dictionary learning for integrative, multimodal and scalable single-cell analysis. Nature Biotechnology (2023). doi: https://doi.org/10.1038/s41587-023-01767-y.

Van Gassen S et al. (2015) FlowSOM: Using self-organizing maps for visualization and interpretation of cytometry data. Cytom Part J Int Soc Anal Cytol 87: 636-645. https://onlinelibrary.wiley.com/doi/full/10.1002/cyto.a.22625.

Aghaeepour, N., Nikolic, R., Hoos, H.H. and Brinkman, R.R. (2011), Rapid cell population identification in flow cytometry data. Cytometry, 79A: 6-13. https://doi.org/10.1002/cyto.a.21007.

Campello, R.J.G.B., Moulavi, D., Sander, J. (2013). Density-Based Clustering Based on Hierarchical Density Estimates. In: Pei, J., Tseng, V.S., Cao, L., Motoda, H., Xu, G. (eds) Advances in Knowledge Discovery and Data Mining. PAKDD 2013. Lecture Notes in Computer Science(), vol 7819. Springer, Berlin, Heidelberg. https://doi.org/10.1007/978-3-642-37456-2_14

Examples

```
obj <- sketch_wrapper(channel = demo_lcmv,
                      meta_data = demo_lcmv,
                      n_sketch_cells = 5000,
                      clst_algorithm = 1,
                      ratio = TRUE,
                      thresholding_method = "otsu")
```

`split_plot_sketch` *Split plot wrapper.*

Description

Dimensional reduction (UMAP) plot split by a given parameter. Per default the returned split plots are rasterized using `geom_point_rast`. Requires ggrastr.

Usage

```
split_plot_sketch(obj, group_by = "seurat_clusters", split_by = "ratio_anno")
```

Arguments

`obj`	The Seurat object.
`group_by`	Parameter to group the plot by.
`split_by`	Parameter to split the plot by.

Value

ggplot object

umap_rasterized *Rasterized UMAP*

Description

Wrapper function to plot a dimensional reduction plot colored by clusters.

Usage

```
umap_rasterized(
  data = obj,
  group.by = "seurat_clusters",
  raster.dpi = 500,
  label = TRUE,
  cols = pals::tol.rainbow(70),
  umap1 = "umap_1",
  umap2 = "umap_2",
  reduction = "umap",
  raster = TRUE
)
```

Arguments

data	Seurat object.
group.by	meta.data column to group the dimensional reduction plot by, for example a clustering solution.
raster.dpi	Pixel resolution (numeric; default 500).
label	Boolean. Plot labels?
cols	Color palette.
umap1	First UMAP dimension as found in the meta.data.
umap2	Second UMAP dimension as found in the meta.data.
reduction	Which reduction to use.
raster	Boolean. Rasterize the plot?

Value

Plot

wrapper_for_plots *Plot wrapper function.*

Description

A wrapper function for plots that are often used during exploratory analysis within the Seurat framework (https://satijalab.org/seurat/). Includes FeaturePlot, DimPlot for different parameters, and ratio_cluster_plot.

Usage

```
wrapper_for_plots(
  obj = obj,
  feature_plot = TRUE,
  cluster_plot = TRUE,
  meta_list = list("ratio_anno"),
  cluster_handle = "sketch_snn_res",
  feature_plot_colors = pals::parula(1000),
  ratio_plot_color = c(Ratio_low = "dodgerblue2", Ratio_high = "gold2"),
  reduction = "umap",
  alpha = 1,
  raster = F,
  label_size = 3,
  label_box = FALSE,
  assay = "sketch"
)
```

Arguments

`obj`	The Seurat object.
`feature_plot`	Boolean. TRUE indicates that UMAP is colored for all features (see `FeaturePlot`)
`cluster_plot`	Boolean. TRUE indicates that UMAP is colored by the different cluster resolutions stored with the cluster_handle.
`meta_list`	List of meta.data columns to color `DimPlot` by. Can be both numeric to generate `FeaturePlot` or class character or factor for `DimPlot`.
`cluster_handle`	Prefix for the clustering solutions in the meta.data slot.
`feature_plot_colors`	Color palette for `FeaturePlot`.
`ratio_plot_color`	Color palette for `ratio_cluster_plot`.
`reduction`	Reduction to use for plotting, for example UMAP.
`alpha`	Alpha value for plotting.
`raster`	Convert points to raster format. If TRUE plot is rasterized to raster.dpi=c(512, 512). Requires ggrastr.
`label_size`	Size of the labels plotted within the embedding.
`label_box`	Plot boxes around labels in the color of the cluster.
`assay`	The Seurat assay (default FACS).

Value

A list with the requested plots.

Index

∗ **datasets**
    demo_lcmv, 3

auto_thresh, *2*

calculateThreshold, 2, *8*

demo_lcmv, 3
DimPlot, *11*, *12*

FeaturePlot, *11*, *12*
FindClusters, *9*
flowMeans, *9*

geom_point_rast, *10*

hdbscan, *9*

lda, *5*

MEM_heatmap, 4

open_matrix_dir, *9*

pdf, *7*
predict_data, 5

ratio_cluster_plot, 6, *7*, *11*, *12*
run.flowsom, *9*

save.obj, 6
save.plot, 7
select_dbt, 7
sketch_wrapper, 8
SketchData, *8*, *9*
split_plot_sketch, 10

umap_rasterized, 11

wrapper_for_plots, 11
