# Supplementary material for: Ultra-high-scale cytometry-based cellular interaction mapping
Source: Nat Methods. 2025 Aug 7;22(9):1887–99. doi: 10.1038/s41592-025-02744-w (PMC12446065; doi:10.1038/s41592-025-02744-w)
Supplement: Supplementary file 5 — PICtR source code repository. [file 41592_2025_2744_MOESM5_ESM.zip › PICtR-main/PICtR_1.0.0.tar.gz!/PICtR/inst/doc/PICtR.html]

PICtR: Physically Interacting Cell toolkit for R


# PICtR: Physically Interacting Cell toolkit for R

#### Viktoria Flore

#### last updated 01/2025

- 1 Introduction
- 2 Installation
- 3 PICtR Pipeline
  - 3.1 Pre-Processing of Cytometry
    Data
  - 3.2 Example Data
  - 3.3 Subsampling, Dimensional Reduction
    and Clustering
  - 3.4 Predict Labels for the full Data
    Set
  - 3.5 Selecting Interacting
    Populations
  - 3.6 Singlet Annotation
  - 3.7 Annotation of Interacting
    Populations
  - 3.8 Downstream Analysis
- 4 Citing PICtR
- 5 References

# 1 Introduction

Many fundamental processes in life are shaped by physical
interactions between cells. PICtR provides a computational workflow to
analyse both cellular landscapes and cellular interactions in
high-dimensional flow cytometry data using R. PICtR can be used with any
cytometry-based assay and the approach can also be applied to existing
datasets, provided that the following guidelines are met:

- The data stems from a case-control setting
- All samples were treated the same during sample processing and
  acquisition, e.g. regarding equal cell concentrations, incubation times,
  and flow rates
- Pooling across conditions, organs or donors should be avoided
- For *in vivo* experiments, cells with a strong affinity to
  interact can artificially interact if they are brought into proximity
  during sample preparation, even if they were physically separated *in
  vivo*. These interactions may still reflect biological effects, but
  this should be taken into account when interpreting the results.

# 2 Installation

PICtR requires R version 4.3 or later. First, please install BPCells. Next, PICtR can be
installed using:

```
if (!require("remotes", quietly = TRUE)) {
  install.packages("remotes")
}

remotes::install_github("agSHaas/PICtR")
```

Some other packages are not necessarily required, but expand the
functionality of PICtR. Consider installing:

- flowMeans
- dbscan
- Spectre
- ComplexHeatmap
- cytoMEM
- autothresholdr

# 3 PICtR Pipeline

## 3.1 Pre-Processing of Cytometry Data

Data from FCS files should be unmixed/compensated and transformed
(for example using FlowJo from BD). We recommend using quality control
tools such as PeacoQC
or FlowAI to
ensure high quality cytometry data. Export the populations of interest
using channel values defined by the inbuilt export function of FlowJo or
use flowWorkspace.

## 3.2 Example Data

We provide a small demo data set from the spleens of lymphocytic
choriomeningitis virus (LCMV) infected mice and controls. Five days
prior to infection, LCMV-specific CD4 (CD90\_1-positive) T cells were
transferred into the hosts. The samples were analysed using high
parametric spectral flow cytometry with *n* = 36 markers,
allowing the detection of all important cell types across the immune
cell landscape. For the demo data set, we will focus on 10 different
markers:

- CD3: T cell marker
- MHCII: Major histocompatibility complex II, expressed on
  antigen-presenting cells
- CD11c: Dendritic cell (DC) marker
- CD11b: Myeloid cell marker
- CD45\_2: Pan-hematopoetic marker, allelic form 2
- CD19: B cell marker
- Ly6G: Neutrophil marker
- CD90\_1: Marker for LCMV-specific CD4 T cells
- CD4: Expressed on CD4 T cells and some myeloid populations
- CD8: Expressed on CD8 T cells, some natural killer cells (NK) and
  some dendritic cells

Details on the experimental setup can be found in Vonficht D,
Jopp-Saile L, Yousefian S, Flore V et al. Ultra-high scale
cytometry-based cellular interaction mapping. *Nature Methods*
(2025).

Demo data can be loaded with `data(demo_lcmv)`. See
`?demo_lcmv` for details.

## 3.3 Subsampling, Dimensional Reduction and Clustering

The pipeline starts with a data frame with the dimensionality cells x
flow cytometry parameters. First, we calculate the FSC ratio based on
the forward scatter area (FSC-A) and forward scatter height (FSC-H)
parameters. The FSC ratio is highly discriminatory for singlets and
multiplets, and will help us to select interacting populations after
clustering. We find a threshold that classifies all events as having a
low or high FSC ratio using thresholding methods such as Otsu
thresholding.

```
library(PICtR)
data("demo_lcmv")
```

```
threshold <- calculateThreshold((demo_lcmv$FSC.A/demo_lcmv$FSC.H), method = "otsu")

hist(demo_lcmv$FSC.A/demo_lcmv$FSC.H, breaks = 2000)
abline(v = threshold)
```

Then, we create a Seurat
object using forward scatter parameters, side scatter parameters, cell
type markers and the FSC ratio as features. Several steps of the PICtR
workflow make use of the Seurat framework.  
We store the counts layer on-disk using BPCells, enabling us to
analyse millions of events while keeping computational time manageable.
Thus far, we have analysed data sets with more than 60 million events
with PICtR.  
In addition, we sample a representative subset of cells using atomic
sketching as implemented in Seurat.
In contrast to random sampling, sketching retains both abundant and rare
cell types, including cellular interactions. For the demo data set, we
sample `n_sketch_cells = 5000` cells. We use the sketched
data set for dimensional reduction and clustering, before predicting
cell type and interaction labels back to the full data set later on.

PICtR provides a convenient wrapper function for these steps,
including calculation of the FSC ratio threshold:

```
demo_obj <- sketch_wrapper(demo_lcmv,
                       meta_data = demo_lcmv,
                       assay = "FACS",
                       FSC.A = "FSC.A", 
                       FSC.H = "FSC.H", 
                       working_dir = getwd(),
                       resolution = c(0.5, 0.8, 1),
                       clst_algorithm = 1,
                       n_sketch_cells = 5000,
                       obj_name = "obj_sketched_non_projected",
                       ratio = T, 
                       thresholding_method = "otsu", 
                       overwrite = T)
#> The BPCells directory will be overwritten
#> Calculation of Ratio
#> Warning: Matrix compression performs poorly with non-integers.
#> • Consider calling convert_matrix_type if a compressed integer matrix is intended.
#> Your newly generated object will be saved under: /private/var/folders/zq/k957_k5s79v97_ptgc729fm1yy94mx/T/RtmpsTjZY1/Rbuild1ba27944f83/PICtR/vignettes/obj_sketched_non_projected.rds
#> 
#> Sketching started...
#> 
#> Finding variable features for layer counts
#> 
#> Calcuating Leverage Score
#> 
#> Attempting to cast layer counts to dgCMatrix
#> 
#> Attempting to cast layer data to dgCMatrix
#> 
#> Sketching is done
#> 
#> Finding variable features for layer counts
#> 
#> Centering and scaling data matrix
#> 
#> PC_ 1 
#> Positive:  CD4, Ly6G, CD11b, CD90-1, CD8, CD11c, SSC.H, MHCII 
#> Negative:  FSC.A, ratio, SSC.A, FSC.H, CD45-2, CD3, CD19 
#> PC_ 2 
#> Positive:  CD4, CD45-2, CD3, CD90-1, CD8, MHCII, CD19, ratio 
#> Negative:  CD11b, SSC.H, Ly6G, SSC.A, FSC.H, CD11c, FSC.A 
#> PC_ 3 
#> Positive:  CD4, CD90-1, CD3, SSC.H, CD11b, SSC.A, FSC.H, CD8 
#> Negative:  MHCII, CD19, ratio, CD45-2, FSC.A, CD11c, Ly6G 
#> PC_ 4 
#> Positive:  CD19, CD90-1, CD4, Ly6G, ratio, FSC.A, SSC.A, MHCII 
#> Negative:  CD11c, CD8, CD45-2, CD11b, CD3, SSC.H, FSC.H 
#> PC_ 5 
#> Positive:  CD11c, MHCII, CD4, CD90-1, CD45-2, SSC.H, CD11b, SSC.A 
#> Negative:  CD8, Ly6G, CD19, CD3, FSC.H, FSC.A, ratio 
#> 
#> Computing nearest neighbor graph
#> 
#> Computing SNN
#> 
#> This message is displayed once every 8 hours.
#> Modularity Optimizer version 1.3.0 by Ludo Waltman and Nees Jan van Eck
#> 
#> Number of nodes: 5000
#> Number of edges: 164643
#> 
#> Running Louvain algorithm...
#> Maximum modularity in 10 random starts: 0.9193
#> Number of communities: 14
#> Elapsed time: 0 seconds
#> Modularity Optimizer version 1.3.0 by Ludo Waltman and Nees Jan van Eck
#> 
#> Number of nodes: 5000
#> Number of edges: 164643
#> 
#> Running Louvain algorithm...
#> Maximum modularity in 10 random starts: 0.8959
#> Number of communities: 16
#> Elapsed time: 0 seconds
#> Modularity Optimizer version 1.3.0 by Ludo Waltman and Nees Jan van Eck
#> 
#> Number of nodes: 5000
#> Number of edges: 164643
#> 
#> Running Louvain algorithm...
#> Maximum modularity in 10 random starts: 0.8813
#> Number of communities: 19
#> Elapsed time: 0 seconds
#> UMAP will return its model
#> 14:02:17 UMAP embedding parameters a = 0.9922 b = 1.112
#> 14:02:17 Read 5000 rows and found 15 numeric columns
#> 14:02:17 Using Annoy for neighbor search, n_neighbors = 30
#> 14:02:17 Building Annoy index with metric = cosine, n_trees = 50
#> 0%   10   20   30   40   50   60   70   80   90   100%
#> [----|----|----|----|----|----|----|----|----|----|
#> **************************************************|
#> 14:02:18 Writing NN index file to temp file /var/folders/zq/k957_k5s79v97_ptgc729fm1yy94mx/T//RtmpDakD95/file1eb15f0ab31
#> 14:02:18 Searching Annoy index using 1 thread, search_k = 3000
#> 14:02:19 Annoy recall = 100%
#> 14:02:19 Commencing smooth kNN distance calibration using 1 thread with target n_neighbors = 30
#> 14:02:19 Initializing from normalized Laplacian + noise (using RSpectra)
#> 14:02:19 Commencing optimization for 500 epochs, with 202814 positive edges
#> 14:02:23 Optimization finished
#> The object will be updated and saved
```

### 3.3.1 Guidelines for Clustering

Finding a good clustering resolution is critical to detecting and
annotating interacting populations. PICtR provides several different
options for clustering: In addition to Louvain, SLM, and Leiden algorithms,
which are implemented in the Seurat framework, FlowSOM, flowMeans and HDBSCAN
(Hierarchical Density-Based Spatial Clustering of Applications with
Noise) are available. We recommend using Louvain or Leiden due to their
successful identification of interacting populations, fast run times,
and easy customization of the resolution parameter (note that Leiden
requires the leidenalg python package).

The effect of the resolution parameter can be seen when comparing a
resolution of 0.5, 0.8 and 1 (Louvain clustering) for the demo data
set:

```
DimPlot(demo_obj, group.by = "sketch_snn_res.0.5", label = T) + theme(legend.position = "none")
DimPlot(demo_obj, group.by = "sketch_snn_res.0.8", label = T) + theme(legend.position = "none")
DimPlot(demo_obj, group.by = "sketch_snn_res.1", label = T) + theme(legend.position = "none")
```

We recommend to over-cluster the data to ensure the detection of
small populations, including cellular interactions. So if you expect
roughly 20 different cell types based on your flow cytometry panel, aim
for a cluster resolution that provides you with more than 20 clusters.
Subsequently, during annotation, clusters representing the same cell
type can be merged. Alternatively, for clusters that represent
populations of interest that require higher resolutions, sub-clustering
can be performed (`FindSubCluster()`). Different resolutions
might be tested before deciding for the best fit.

It can also be helpful to compare your cluster boundaries to feature
plots of the markers used for annotation to see if you capture all
relevant patterns. For our demo data set, a resolution of 0.5 seems to
be sufficient:

```
FeaturePlot(demo_obj, features = c("CD3", "MHCII", "CD11c", "CD11b", 
                                   "CD45_2", "CD19", "Ly6G", 
                                   "CD90_1", "CD4", "CD8", "ratio"))
```

The Seurat tutorials and
Heumos et
al. (2023) provide excellent guidance on clustering of single-cell
data sets.

## 3.4 Predict Labels for the full Data Set

Currently, our cluster labels only exist for the sketched subset of
cells, but we can predict the labels for the entire data set using
linear discriminant analysis (LDA) as implemented in the
`predict_data()` function:

```
demo_obj <- predict_data(obj = demo_obj, 
                      data_query = demo_obj, 
                      ref_clusters = "sketch_snn_res.0.5",
                      pred_name = "clusters_predicted",
                      assay_ref = "sketch", 
                      assay_query = "FACS")
#> Warning: Converting to a dense matrix may use excessive memory
#> This message is displayed once every 8 hours.
```

## 3.5 Selecting Interacting Populations

Now we can select the clusters that represent interacting cells. For
each cluster, we determine the proportion of cells above and below the
FSC ratio threshold that we calculated earlier:

```
ratio_cluster_plot(demo_obj, clusters = "clusters_predicted")
```

As seen from the bar plot, clusters 3, 7, 10, 11, and 13
predominantly contain cells that exceed the FSC ratio threshold. The
`select_dbt()` function can be used to pick these clusters.
We recommend investigating the `ratio_cluster_plot()` to
confirm any selection you make.

## 3.6 Singlet Annotation

Based on the previous analysis, clusters 3, 7, 10, 11 and 13 have
been identified as representing interacting cells. Now, we can annotate
the remaining clusters with the help of the feature plots (see above) or
ridge plots:

```
Idents(demo_obj) <- "clusters_predicted"
RidgePlot(demo_obj, features = Features(demo_obj)) & theme(axis.text = element_text(size = 7))
#> Picking joint bandwidth of 23.7
#> Picking joint bandwidth of 20.3
#> Picking joint bandwidth of 9.11
#> Picking joint bandwidth of 15.5
#> Picking joint bandwidth of 24.9
#> Picking joint bandwidth of 21.7
#> Picking joint bandwidth of 26.5
#> Picking joint bandwidth of 32.7
#> Picking joint bandwidth of 28.4
#> Picking joint bandwidth of 43
#> Picking joint bandwidth of 28.9
#> Picking joint bandwidth of 11.3
#> Picking joint bandwidth of 19.9
#> Picking joint bandwidth of 9.44
#> Picking joint bandwidth of 19.1
```

Based on the marker expression, we can annotate all clusters as
follows:

```
demo_obj@meta.data <- demo_obj@meta.data %>% 
  mutate(celltype = case_when(clusters_predicted == "0" ~ "B cells", 
                              clusters_predicted == "1" ~ "CD8 T cells", 
                              clusters_predicted == "2" ~ "DCs", 
                              clusters_predicted == "3" ~ "interacting cells", 
                              clusters_predicted == "4" ~ "LCMV-spec CD4 T cells", 
                              clusters_predicted == "5" ~ "Myeloid cells", 
                              clusters_predicted == "6" ~ "CD4 T cells", 
                              clusters_predicted == "7" ~ "interacting cells", 
                              clusters_predicted == "8" ~ "Myeloid cells", 
                              clusters_predicted == "9" ~ "Neutrophils",
                              clusters_predicted == "10" ~ "interacting cells", 
                              clusters_predicted == "11" ~ "interacting cells", 
                              clusters_predicted == "12" ~ "DCs", 
                              clusters_predicted == "13" ~ "interacting cells"))

DimPlot(demo_obj, group.by = "celltype", label =T)
```

## 3.7 Annotation of Interacting Populations

Annotating the interacting cells is easier if we subset and
re-cluster them:

```
# subset interacting cells
DefaultAssay(demo_obj) <- "FACS"
Idents(demo_obj) <- "celltype"
interact_obj <- subset(demo_obj, idents = "interacting cells")

# repeat the Seurat workflow 
n_dims <- length(Features(demo_obj))
interact_obj <- interact_obj %>%
  FindVariableFeatures() %>%
  ScaleData() %>%
  RunPCA(npcs=n_dims, approx=F) %>%
  FindNeighbors(dims = 1:n_dims) %>% 
  FindClusters(resolution = c(0.5, 0.8, 1)) %>% 
  RunUMAP(dims = 1:n_dims, return.model = T)
#> Finding variable features for layer counts
#> PC_ 1 
#> Positive:  MHCII, CD19, CD45-2, CD4, Ly6G, CD8, CD90-1, CD11b 
#> Negative:  SSC.A, FSC.A, SSC.H, FSC.H, CD3, CD11c, ratio 
#> PC_ 2 
#> Positive:  CD4, CD3, CD8, CD11c, CD90-1, CD11b, FSC.H, CD45-2 
#> Negative:  CD19, MHCII, SSC.A, ratio, FSC.A, SSC.H, Ly6G 
#> PC_ 3 
#> Positive:  CD45-2, CD4, CD3, ratio, CD19, FSC.A, CD90-1, MHCII 
#> Negative:  Ly6G, CD11b, SSC.H, SSC.A, CD11c, FSC.H, CD8 
#> PC_ 4 
#> Positive:  CD90-1, FSC.H, SSC.H, CD4, FSC.A, SSC.A, CD19, MHCII 
#> Negative:  CD45-2, CD11b, CD11c, CD8, CD3, ratio, Ly6G 
#> PC_ 5 
#> Positive:  CD8, FSC.A, CD11c, FSC.H, ratio, SSC.A, CD19, MHCII 
#> Negative:  CD4, CD45-2, Ly6G, CD90-1, CD11b, CD3, SSC.H
#> Computing nearest neighbor graph
#> Computing SNN
#> UMAP will return its model
#> 14:02:36 UMAP embedding parameters a = 0.9922 b = 1.112
#> 14:02:36 Read 9808 rows and found 15 numeric columns
#> 14:02:36 Using Annoy for neighbor search, n_neighbors = 30
#> 14:02:36 Building Annoy index with metric = cosine, n_trees = 50
#> 0%   10   20   30   40   50   60   70   80   90   100%
#> [----|----|----|----|----|----|----|----|----|----|
#> **************************************************|
#> 14:02:36 Writing NN index file to temp file /var/folders/zq/k957_k5s79v97_ptgc729fm1yy94mx/T//RtmpDakD95/file1eb62f61dfc
#> 14:02:36 Searching Annoy index using 1 thread, search_k = 3000
#> 14:02:38 Annoy recall = 100%
#> 14:02:38 Commencing smooth kNN distance calibration using 1 thread with target n_neighbors = 30
#> 14:02:39 Initializing from normalized Laplacian + noise (using RSpectra)
#> 14:02:39 Commencing optimization for 500 epochs, with 406056 positive edges
#> 14:02:46 Optimization finished
```

```
# plot the different clustering resolutions
DimPlot(interact_obj, group.by = "FACS_snn_res.0.5", label = T) + theme(legend.position = "none")
DimPlot(interact_obj, group.by = "FACS_snn_res.0.8", label = T) + theme(legend.position = "none")
DimPlot(interact_obj, group.by = "FACS_snn_res.1", label = T) + theme(legend.position = "none")
#> Modularity Optimizer version 1.3.0 by Ludo Waltman and Nees Jan van Eck
#> 
#> Number of nodes: 9808
#> Number of edges: 329874
#> 
#> Running Louvain algorithm...
#> Maximum modularity in 10 random starts: 0.8902
#> Number of communities: 11
#> Elapsed time: 0 seconds
#> Modularity Optimizer version 1.3.0 by Ludo Waltman and Nees Jan van Eck
#> 
#> Number of nodes: 9808
#> Number of edges: 329874
#> 
#> Running Louvain algorithm...
#> Maximum modularity in 10 random starts: 0.8573
#> Number of communities: 15
#> Elapsed time: 0 seconds
#> Modularity Optimizer version 1.3.0 by Ludo Waltman and Nees Jan van Eck
#> 
#> Number of nodes: 9808
#> Number of edges: 329874
#> 
#> Running Louvain algorithm...
#> Maximum modularity in 10 random starts: 0.8421
#> Number of communities: 20
#> Elapsed time: 0 seconds
```

```
FeaturePlot(interact_obj, features = c("CD3", "MHCII", "CD11c", "CD11b", 
                                   "CD45_2", "CD19", "Ly6G", 
                                   "CD90_1", "CD4", "CD8", "ratio"))
```

We can move forward with a resolution of 0.5, but there is a pattern
of CD8 within cluster 4 that is not well represented in this clustering
resolution. We can sub-cluster this population on the same
shared-nearest-neighbor graph (`FACS_snn`) using
`FindSubCluster()` with a low resolution:

```
Idents(interact_obj) <- "FACS_snn_res.0.5"
interact_obj <- FindSubCluster(interact_obj, cluster = "4", 
                               graph.name = "FACS_snn", resolution = 0.25)

# plot 
DimPlot(interact_obj, group.by = "sub.cluster", label = T)
#> Modularity Optimizer version 1.3.0 by Ludo Waltman and Nees Jan van Eck
#> 
#> Number of nodes: 845
#> Number of edges: 29301
#> 
#> Running Louvain algorithm...
#> Maximum modularity in 10 random starts: 0.8506
#> Number of communities: 3
#> Elapsed time: 0 seconds
```

Now we can annotate the interacting cells based on mutually exclusive
marker expression. CD19 for example is only expressed on B cells, while
CD3 and CD4 expression is found on CD4+ T cells. When CD19, CD3 and CD4
are simultaneously expressed within an interacting cluster, we know that
the interaction partners are B cells and CD4+ T cells. We will label
interactions with an asterisk (\*):

```
Idents(interact_obj) <- "sub.cluster"
RidgePlot(interact_obj, features = Features(interact_obj)) & 
  theme(axis.text = element_text(size = 7))
#> Picking joint bandwidth of 18.7
#> Picking joint bandwidth of 19.4
#> Picking joint bandwidth of 7.69
#> Picking joint bandwidth of 16.2
#> Picking joint bandwidth of 21.4
#> Picking joint bandwidth of 15.8
#> Picking joint bandwidth of 24
#> Picking joint bandwidth of 30.3
#> Picking joint bandwidth of 23.8
#> Picking joint bandwidth of 47.8
#> Picking joint bandwidth of 28
#> Picking joint bandwidth of 9.23
#> Picking joint bandwidth of 14.4
#> Picking joint bandwidth of 6.68
#> Picking joint bandwidth of 21.2
```

```
interact_obj@meta.data <- interact_obj@meta.data %>% 
  mutate(interact_type = case_when(sub.cluster == "0" ~ "B cells only", 
                              sub.cluster == "1" ~ "B cells only", 
                              sub.cluster == "2" ~ "B*DC", 
                              sub.cluster == "3" ~ "B*CD4 T", 
                              sub.cluster == "4_0" ~ "DC*LCMV-spec CD4 T", 
                              sub.cluster == "4_1" ~ "B*LCMV-spec CD4 T", 
                              sub.cluster == "4_2" ~ "B*DC*LCMV-spec CD4 T", 
                              sub.cluster == "5" ~ "CD4 T*DC", 
                              sub.cluster == "6" ~ "B*CD8 T", 
                              sub.cluster == "7" ~ "B*Neutrophils", 
                              sub.cluster == "8" ~ "CD4*CD8", 
                              sub.cluster == "9" ~ "B*Myeloid",
                              sub.cluster == "10" ~ "remove"))
```

Cluster 10 has a lower CD45\_2 expression and a low ratio, so we will
remove it from the interacting landscape. Clusters 0 and 1 do not
exhibit mutually exclusive marker expression - both populations only
express B cell markers. It is likely that they represent homotypic B
cell interactions, but we cannot rule out alternative explanations such
as preceding cytokinesis or interactions with cells that we do not have
a marker for. So we will exclude these populations and recalculate the
UMAP embedding:

```
keep <- interact_obj@meta.data %>% 
  dplyr::filter(interact_type %in% c("B*Neutrophils", 
                                     "CD4*CD8", 
                                     "B*CD4 T",             
                                     "B*Myeloid",
                                     "B*DC",
                                     "DC*LCMV-spec CD4 T",
                                     "B*LCMV-spec CD4 T",
                                     "B*DC*LCMV-spec CD4 T",
                                     "B*CD8 T",
                                     "CD4 T*DC"))
interact_obj <- subset(interact_obj, cells = rownames(keep))

interact_obj <- interact_obj %>% 
  RunUMAP(dims = 1:n_dims, return.model = T)
#> UMAP will return its model
#> 14:02:51 UMAP embedding parameters a = 0.9922 b = 1.112
#> 14:02:51 Read 5482 rows and found 15 numeric columns
#> 14:02:51 Using Annoy for neighbor search, n_neighbors = 30
#> 14:02:51 Building Annoy index with metric = cosine, n_trees = 50
#> 0%   10   20   30   40   50   60   70   80   90   100%
#> [----|----|----|----|----|----|----|----|----|----|
#> **************************************************|
#> 14:02:52 Writing NN index file to temp file /var/folders/zq/k957_k5s79v97_ptgc729fm1yy94mx/T//RtmpDakD95/file1eb14da0c39
#> 14:02:52 Searching Annoy index using 1 thread, search_k = 3000
#> 14:02:53 Annoy recall = 100%
#> 14:02:53 Commencing smooth kNN distance calibration using 1 thread with target n_neighbors = 30
#> 14:02:53 Initializing from normalized Laplacian + noise (using RSpectra)
#> 14:02:53 Commencing optimization for 500 epochs, with 222518 positive edges
#> 14:02:57 Optimization finished
```

```
# plot 
DimPlot(interact_obj, group.by = "interact_type", label = T)
```

## 3.8 Downstream Analysis

After annotating the singlet and interacting landscapes, you can
continue with downstream analyses according to your experimental setup.
Often, this will involve the quantification of interactions in the case
and control conditions to test for significant differences.

# 4 Citing PICtR

If you are using PICtR in your research, please consider citing:

- Vonficht D, Jopp-Saile L, Yousefian S, Flore V et al. “Ultra-high
  scale cytometry-based cellular interaction mapping”, *Nature
  Methods* (2025)

# 5 References

- Vonficht D, Jopp-Saile L, Yousefian S, Flore V et al. Ultra-high
  scale cytometry-based cellular interaction mapping. *Nature
  Methods* (2025).
- Hao Y et al. Dictionary learning for integrative, multimodal and
  scalable single-cell analysis. *Nat Biotechnol* 42, 293–304
  (2024). https://doi.org/10.1038/s41587-023-01767-y
- Heumos L, Schaar A.C. et al. Best practices for single-cell analysis
  across modalities. *Nat Rev Genet* (2023). https://doi.org/10.1038/s41576-023-00586-w
- Parks B & Abdi I. BPCells: Single Cell Counts Matrices to PCA. R
  package version 0.2.0 (2024) https://bnprks.github.io/BPCells
